# Law of coal caving behind the flexible shield support in pseudo-inclined working face

**DOI:** 10.1371/journal.pone.0261355

**Published:** 2021-12-30

**Authors:** Qinjian Zhan, Niaz Muhammad Shahani, Zhicheng Xue, Shengqiang Li

**Affiliations:** 1 Architectural Engineering Institute, Guangdong University of Petrochemical Technology, Maoming, Guangdong, China; 2 School of Mines, China University of Mining and Technology, Xuzhou, Jiangsu, China; 3 The State Key Laboratory for Geo Mechanics and Deep Underground Engineering, China University of Mining and Technology, Xuzhou, Jiangsu, China; Stellenbosch University, SOUTH AFRICA

## Abstract

Complex boundary conditions are the major influencing factors of coal caving law in the pseudo-inclined working face. The main purpose of this study is to analyze coal caving law of flexible shield support and then to establish the internal relations among coal caving parameters under complex boundary conditions. Firstly, the law of coal caving in different falling modes is simulated physically. Secondly, the coal caving shape, displacement field, and contact force field is simulated. Then, coal caving law and process parameters is analyzed theoretically. Finally, the test was performed in Bai-Ji Mine. The research shows that ellipsoidal ore drawing theory has universal applicability in coal drawing law analysis and parameter optimization. After the Isolated Extraction Zone and Isolated Movement Zone reach the roof, the expansion speed is marked by a short delay, and then, while expanding to the floor, two butted incomplete ellipsoids are formed. There is a time-space difference in coal caving after the support, and some coal will be mined in the next round of coal caving. There are obvious differences in the coal loosening range, displacement field, and contact force field on both sides of the long axis. When the support falls along with the bottom plate, it is more conducive to the release of coal. The test shows that the research is of great significance for optimizing the caving parameters of flexible shield support in the pseudo-inclined working face of the steep seam.

## Introduction

Only by understanding the basic rules of draw, namely, the gravity flow of fragmented ore and rock during the draw process, the caving method can be applied efficiently, and the ore loss and dilution can be decreased to the greatest degree [1–3]. Based on the continuity hypothesis, the density field and the velocity field of flowing particles are regarded as continuous functions, and then the establishment of ore drawing model is often the basis of drawing theory. Like other theories [4, 5], through theoretical analysis, physical simulation [1], numerical simulation [6, 7], and industrial experiments, the existing ore drawing theories mainly include: Ellipsoid ore-drawing theory, ellipsoid-like ore-drawing theory, random medium ore-drawing theory and Bergmark-Roos ore-drawing theory, etc. Among them, based on a large number of experimental studies, the morphology of IEZ (Isolated Extraction Zone, the original location of material that has been extracted from the model) and IMZ (Isolated Movement Zone, the original location of material that has moved in response to the aforementioned extraction) are regarded as an ideal rotating ellipsoid, and the ellipsoid ore drawing theory has developed into one of the more perfect ore drawing theories [8, 9].

Since 1938, when the view that the shape of the released body is ellipsoid, many scholars have been conducted in-depth research [4, 10]. The initial ellipsoid mathematical equation and the velocity equation were based on two assumptions. One is the case that the relative height of particles on the surface of the emitted body remains unchanged during the process of movement. The second is the case that the loose particles are ideal particles (quadratic looseness coefficient is 1.0) [11]. Based on the transition relationship, the mathematical equation of the uniform-eccentricity body and the velocity equation are established, and the internal relations among the parameters are analyzed, including: the body of the uniform eccentricity, the loose body, the uniform velocity body, the descending funnel and the velocity of migration [12]. Furthermore, Li et al. established a unified mathematical equation based on eccentricity equation 1-*ε*^2^ = *KH*^-n^, an ellipsoid ore drawing theory with variable eccentricity is formed. The theory preserves the reasonable parts of the isoeccentric ellipsoid, such as trace equation, transition equation, correlation relation, etc. For example, the shape of the outliers is ellipsoid and the distribution of the velocity field is characteristic [13]. In addition, the necessity of introducing the velocity retardation coefficient into the density equation and the velocity equation is also demonstrated.

In addition, as the improvement and development of ellipsoid ore drawing theory has progressed, some scholars have tried to give other forms of mathematical equations and speed equations. For example, based on the mathematical equation of the expected volume, Gao et al. demonstrated the ore drawing process and the transition relationship, and the relationship between the ore drawing process and the transition is demonstrated [14]; Wang et al. established the theory of random medium ore drawing [15]; On the basis of studying the distribution probability of moving particles, Ren et al. established the mathematical equations of moving funnel, particle size and coordinate transformation by establishing the density equation of the probability of moving particles [16].

Carrying out similar material simulation experiments is convenient for observing the movement law of particles, and can reflect the true form of the released body and the loose body [17]. It is necessary in order to explore the process of ore dilution and verify the rationality of the theoretical model. Moreover, the simulation experiment of similar materials can provide more direct reference value for adjusting stope structure and determining ore drawing plan. Nearly a century ago, scholars have carried out physical simulation experiments on the law of ore drawing. For example, Lehman and McNicholas studied the recovery rate of Miami copper mine and Climax molybdenum mine respectively [18, 19].

Thus far, many physical simulation experiments are used to study the release law of loose particles. For example, Zhang, Tao, and Zhu carried out physical simulation experiments to explore the influence of ore bed dip angle and thickness on the shape of drawing body [20]. Chen et al. has conducted the physical simulation experiments to study the influence of flexible shield support, drawing a point location, and particle density of the additional stress of drawing body [5]. Wang and Zhang et al. found that the shields of the hydraulic support not only affected the volume of the discharging body, but also caused the deflection of the discharging body [21]. Chen and Xiao through the study of multi-point ore drawing, pointed out that the ore drawing is positively correlated with the ore drawing height at first and then linearly correlated [22]. In the past few years, an experimental result based on the largest three-dimensional physical model in the world is undoubtedly encouraging, the shape and size of the ore drawing hole have little influence on the shape of the body, and the geometric similarity constant has little influence on the shape of the body [23]. This indicates that it is possible to scale the shape of the outliers obtained from the experiment in similar physical models, including full scale. At the same time, it also shows that the physical simulation experiment has a high reference significant and applicable value for the study of ore drawing law.

The reasonable numerical calculation method has a guiding role for experimental research and theoretical analysis. Whether setting different load paths, constructing complex boundary conditions, collecting and processing simulation data, or in parameter optimization, effect prediction, cost-saving, and other aspects, the auxiliary role of numerical simulation technology reflects the important value, which is more and more favored by many researchers [24–26]. PFC software developed by ITASCA Company based on discrete element theory can intuitively display the trace of particles, ore drawing process, back coal morphology, and ore rock mixing process, which has helped many researchers obtain the significant research results. Pierce and Bridgewater reveal the seepage process of fine particles in the process of ore drawing through simulation, and give the calculation method of velocity according to the simulation results [27]. Through numerical simulation, Liu et al. carried on the optimization research on the technological parameters of the non-pillar mining face [28]. Sun et al. established a three-dimensional model to study the caving process under different boundary conditions [29].

**Fig 1:**
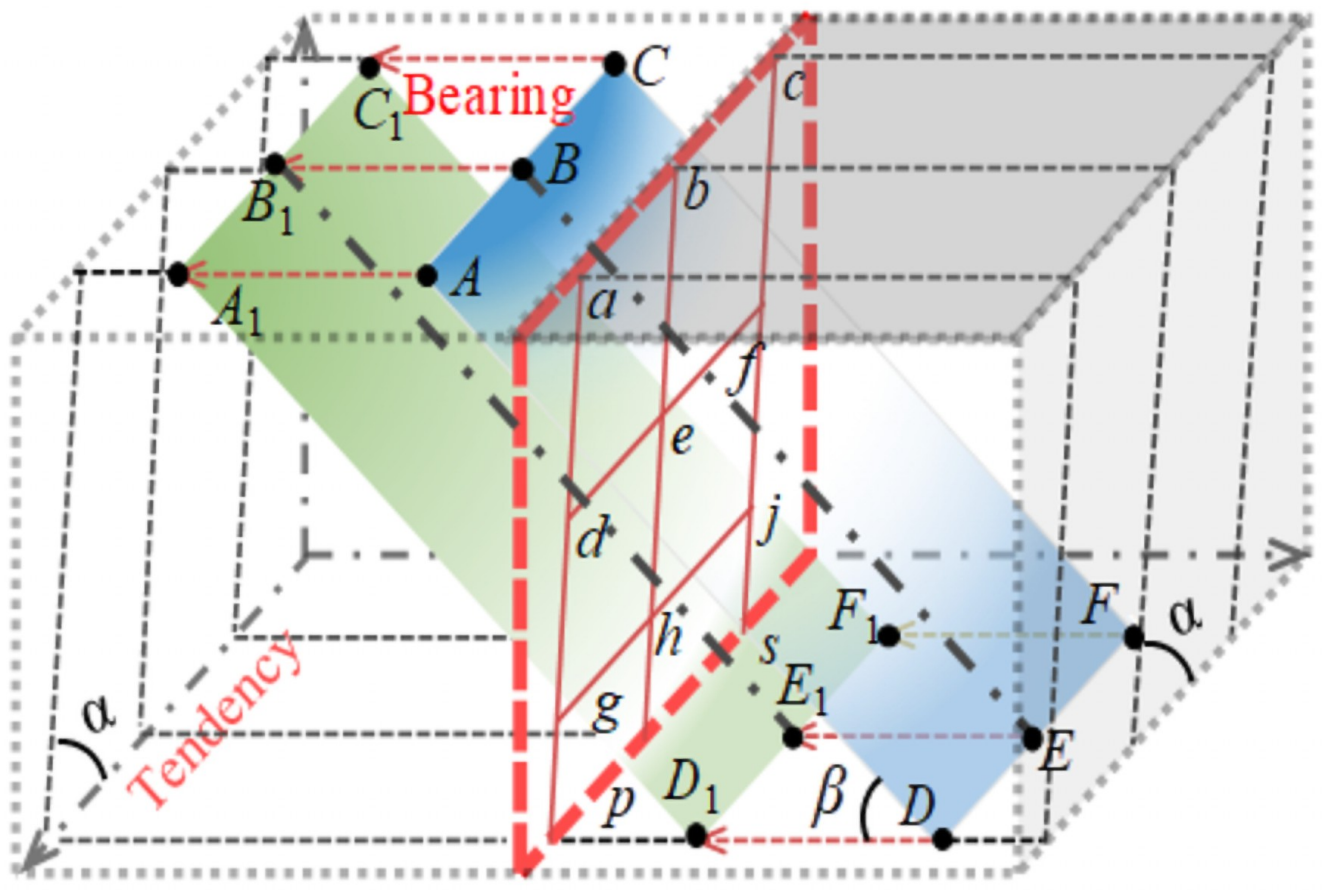
Regionalization of the pseudo-inclined working face.

To sum up, many research results focusing on the ore drawing law under infinite or half boundary (vertical) conditions have greatly promoted the development of ore drawing theory. However, there is still a lack of comprehensive understanding of the law of ore drawing under the condition of complex boundary (inclining), such as the drawing law of coal from the pseudo-inclining working face of steeply inclined coal seam (hereinafter referred to as "working face"). As shown in Fig 1, plane *A*_1_*D*_1_*DA* is the interface between the coal seam and roof, *C*^1^*F*_1_*FC* is the interface between the coal seam and floor, *α* is the seam inclination angle, and *β* is the pseudo-inclination angle of working face. The support is placed along the working face in a direction parallel to *AB*, and the working face advances along the coal seam. The coal body in hexahedron *B*_1_*A*_1_*D*_1_*E*_1_*BADE* is generally mined through blasting and then filled with gangue caved by the roof, which is defined as the front hillock-zone (*□degh*), the coal body in hexahedron *C*_1_*B*_1_*E*_1_*F*_1_*CBEF* is the coal body to be discharged (*□efhg*), the upper part of the plane *ACDF* is the hillock, defined as the top hillock-zone (*□acdf*), and the coal body in the lower part of the plane *A*_1_*C*_1_*D*_1_*F*_1_ is the coal body to be mined (*□gjps*).

**Fig 2:**
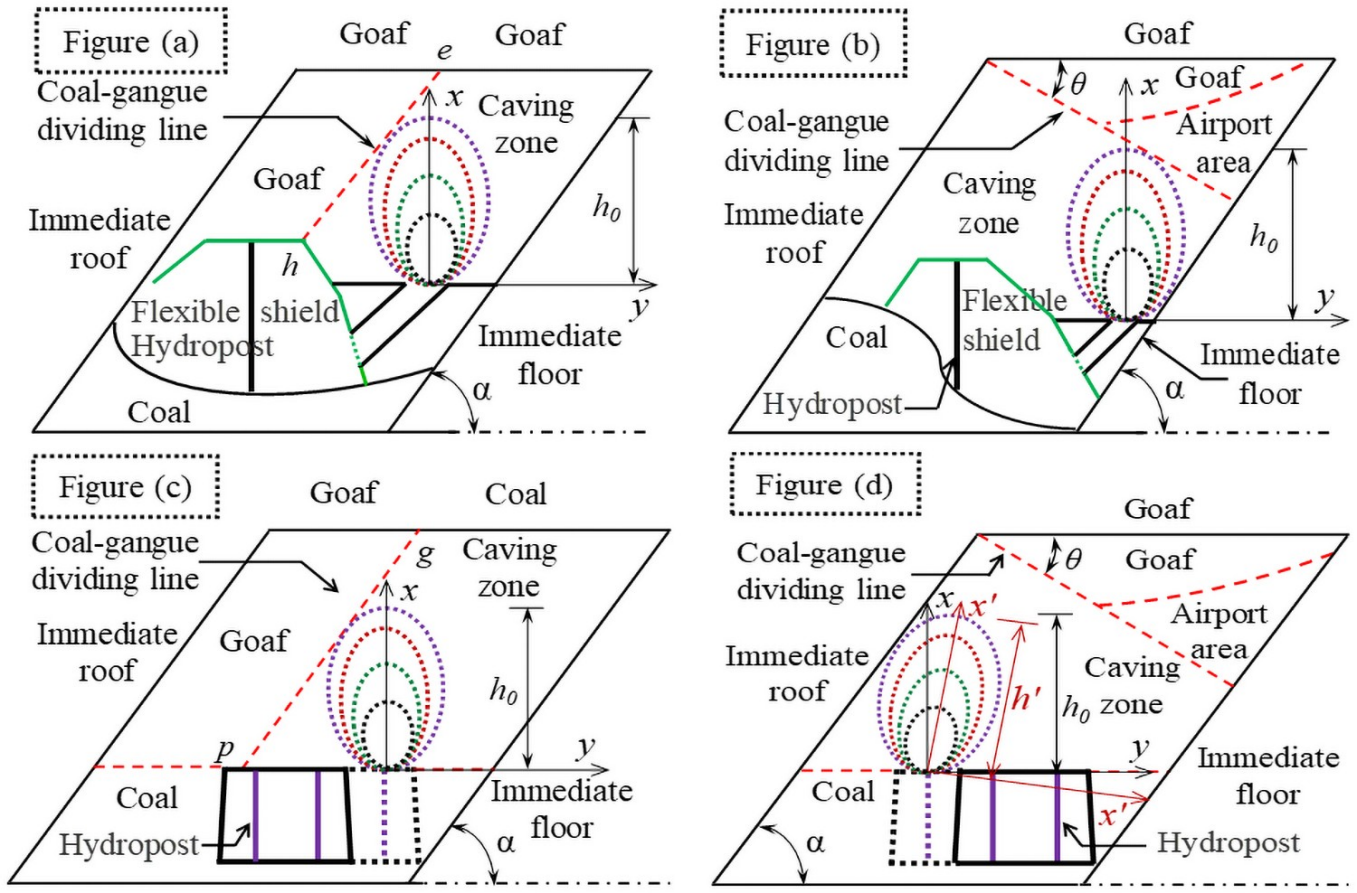
Complex boundary conditions and coal caving forms. As shown in Fig (a), law of coal body release is mainly affected by the front gangue, roof gangue, structure form of support and floor. As shown in Fig (b), when the support falls, the coal body falls immediately, due to the clamping effect of the rock layer, the "fracture-falling-breaking" process of the roof lags behind the coal body and forms an temporary overhead area with the fallen coal body, *θ* is the natural angle of repose of coal, and also the inclination of the interface between coal and gangue, when the support puts coal, the airport area will gradually disappear. Therefore, law of coal body release is mainly affected by the top gangue, the structure form of support and the roof. As shown in Fig (c), law of coal body drawing is mainly affected by the front gangue and floor. As shown in Fig (d), the coal body drawing law is mainly affected by the roof, top gangue and floor.

According to Fig 1, compared with ordinary top coal, the coal to be discharged is located on the flank of the working face. According to the coal seam inclination angle, the support structure, vertical height of the section and the coal discharge position, the flexible shield support coal discharge can be roughly divided into four basic forms: the working face support drops coal along the roof, the working face drops coal along the floor, and the flexible shielding support (hereinafter referred to as "tail frame") for transporting the flat roadway tail puts down along the roof, and the tail support drops coal along the floor. The basic form of coal caving after support under complex boundary conditions is shown in Fig 2.

Thus it can be seen that, the drawing law of coal behind the support has certain particularity. This is explained by the fact that, under the influence of boundary conditions, although there is a transition relationship between particles on the surface of ellipsoid in the process of movement, the overall trend of these particles reaching the ore drawing outlet at the same time is still obvious. When coal and gangue interface CGDF (Coal-gangue dividing face) is tangent to each other, the continuous coal caving will bring the dilution phenomenon. Therefore, the complex boundary conditions should be part of the main factors affecting the law of coal body drawing behind the flexible shield support in pseudo-inclined working face of steep coal seam.

Based on the self-designed experimental platform, this paper systematically studied the law of the coal body behind the flexible shield support through theoretical analysis, physical simulation, numerical simulation and industrial test, and discussed the influence of falling mode and boundary conditions on the drawing law of the coal body. On this basis, the internal relations among the parameters, such as the distance of coal caving, the ratio of drawing to cave and the thickness of coal seam, are established. The results show that the theory of ellipsoid ore drawing has general adaptability in the analysis of the law of coal body drawing and the optimization of coal drawing process parameters. The particularity of the law of coal body releasing lies in the different boundary conditions that prevent the shape expanding of the releasing body and the loose body. The successful practice of coal mining with flexible shield supports at Bai-Ji Mine not only provides a successful case for coal drawing under similar geological conditions, but also points out the direction of this type of technology to overcome difficulties.

## Methods and materials

The research mainly focusses on the self-designed experimental platform, and the flexible shield support is selected for the working face. In order to ensure the similarity between the on-site coal caving and the simulated coal caving in the laboratory, the geometric similarity ratio of the preparation of the experimental platform and the laying of the model is 1:20, the production process and similar materials are selected as follows:

1. The support is made of stainless steel, the size is 1072 mm × 777 mm × 500 mm, the coal seam inclination and the pseudo-inclination of the working face can be adjusted by the installed lever. In order to realize the visualization of coal drawing law, Aleck glass plate was used to replace the roof and the boundary of the model.
2. The support of the working surface is made of galvanized iron sheet and fixed on the triangular wooden support with wooden board and iron wire. According to the similarity theory, the width of the iron sheet is 175 mm, and a gap of 15 mm×15 mm is arranged on it as the coal discharge port.
3. The tail support is mainly composed of boards and wooden supports, the boards are sample solid boards with a thickness of 10 mm. The gaps of 20 mm × 20 mm are spaced on the axis of the boards to simulate the release of coal behind the supports.

The experimental models are mainly classified into two types: the first is the coal discharge model of working face support; the second is the tailstock coal discharge model. The dip angle of the coal seam is 50°, 70° and 90° respectively. The vertical height difference of working face is 10.0 m, and the pseudo dip angle is 25°. The similar material is quartz sand, the coal seam is laid with a thickness of 5.0 m, and the marker layer with a spacing of 1.0 m is coarse-grained rice and dyed quartz sand. The physical and mechanical parameters of coal and gangue are listed in Table 1.

**Table 1:** Physical parameters of coal and gangue.

Rock mass	Density / kg·m^-3^	Normal stiffness / kN·m^-1^	Shear stiffness / kN·m^-1^	Frictional coefficient	Repose angle / °	Cohesive force / N
Coal	1400	2.0×10^5^	2.0×10^5^	0.40	50	0
Gangue	2650	4.0×10^5^	4.0×10^5^	0.40	50	0

## Schemes and results

### Caving rule of haulage roadway

#### On the strike profile

**Fig 3:**
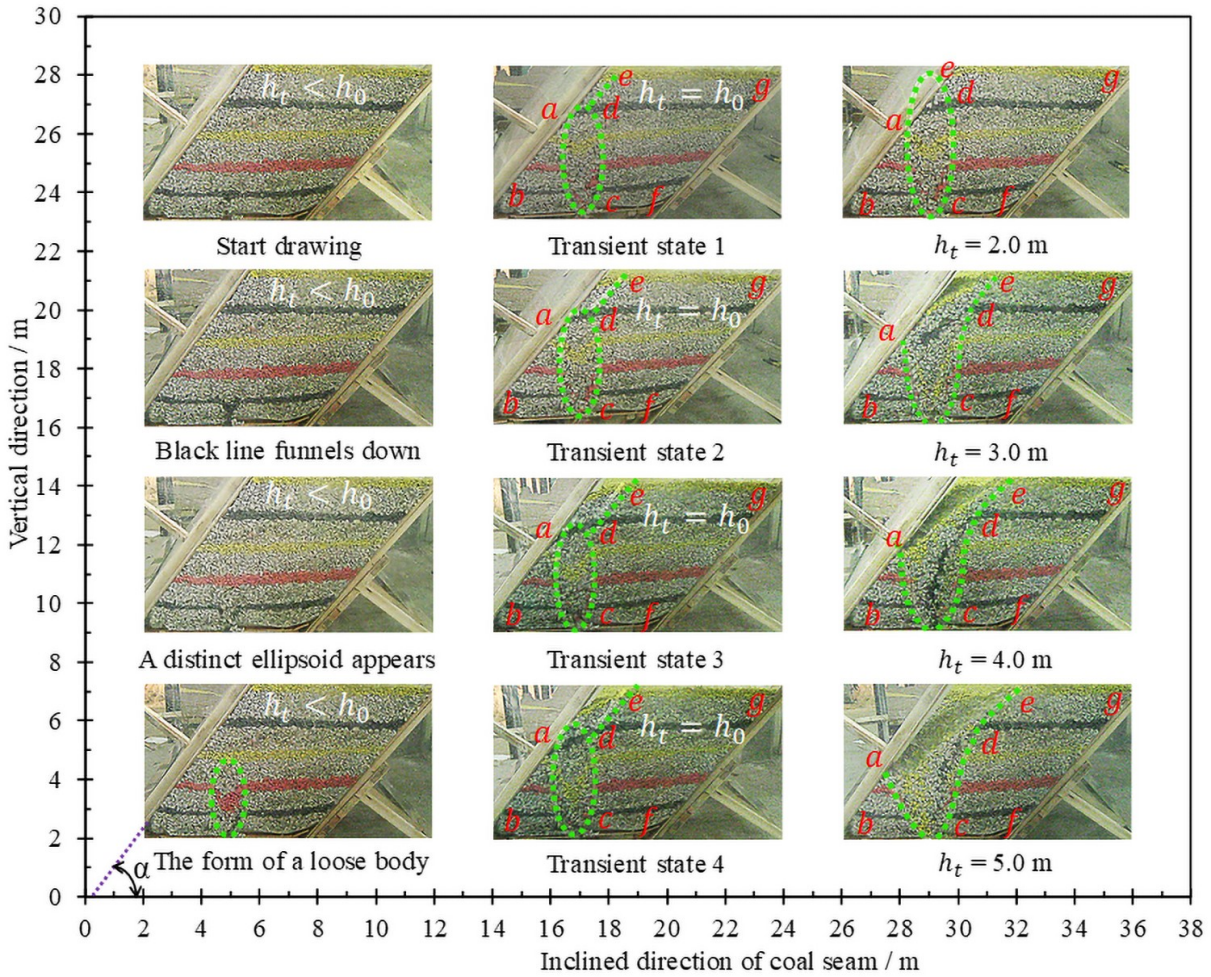
Loose form and discharging process of coal.

The analysis process follows the principle that the loose range is the released range [30, 31]. Suppose the loosening height of coal body at any time is *h*_t_, the vertical distance from coal discharge port to roof is *h*_0_, the part below *h*_0_ is defined as lower coal body, and the part above *h*_0_ is defined as upper coal body. The process of releasing the coal behind the tailstock is shown in Fig 3. The loosening and discharging of coal body show obvious timeliness:

1. When *h*_t_<*h*_0_, both the loosening body and the releasing body expand in the form of an ellipsoid. When *h*_t_ = 1.0 m, the long and short semi-axes of the loose ellipsoid are 1.35 m and 0.62 m respectively. When *h*_t_ = 2.0 m, it’s long and short semi-axes are 2.36 m and 0.75 m respectively. Based on the statistical analysis of multiple experimental data, the experimental constants related to this simulation of similar materials are: *m* = 0.78, *n* = 1.32, respectively.
2. When *h*_t_ = *h*_0_, the loose ellipsoid extends to the boundary of the roof, and the loose range extends upward along the boundary of the roof until a stable sliding surface is formed, and the long and short axes of the loose ellipsoid have almost no growth. The analysis shows that before the formation of slip surface, the upper coal body will continuously slide to the ellipsoid, which leads to a certain lag in the expansion of the lower loose ellipsoid compared with the expansion of the upper range loose of coal body.
3. When *h*_t_>*h*_0_, the slip plane gradually expanded to the top of the model, during which the incline angle of the slip plane was almost maintained at about 50°. The loose ellipsoid then evolved into a gradually expanding broken ellipsoid. When the coal is continuously discharged, the slip surface will gradually loosen and fall the coal body in a manner of gradually moving towards the floor.

There are obvious regional differences in the drawing and loosening of coal body:

Along the strike direction, the projection area and release rate of each part of the coal body behind the support are listed in Table 2. When 1.0 m≤*h*_t_≤2.0 m, the loosening range has not been extended to the roof. As the loosening ellipsoid is gradually expanded, the area of the triangle (*abc*) and the polygon (*edcfg*) decreases obviously. When 2.0 m≤*h*_t_≤4.0 m, the area of the triangle is reduced slightly, while the area of the polygon is still significantly reduced. It shows that when the loosening range extends to the roof boundary, the lower coal body loosens more slowly, while the upper coal body loosens more obvious. When 4.0 m≤*h*_t_≤5.0 m, the triangle area is obviously reduced, indicating that the loose range of the lower coal body is gradually expanding.

**Table 2:** Projection area and discharge rate of each part of the coal body.

Release height /m	Triangle *abc*		Polygons *edcfg*		Outgoing triangle	
	Surface / m^2^	Percent / %	Surface / m^2^	Percent / %	Surface / m^2^	Percent / %
1	6.02	18.46	26.61	81.54	0	0
2	4.60	14.08	25.34	77.66	0	0
3	4.44	13.61	21.23	65.04	0.67	2.06
4	4.25	13.04	19.98	61.21	2.15	6.60
5	3.52	10.79	18.73	57.38	3.74	11.45

**Fig 4:**
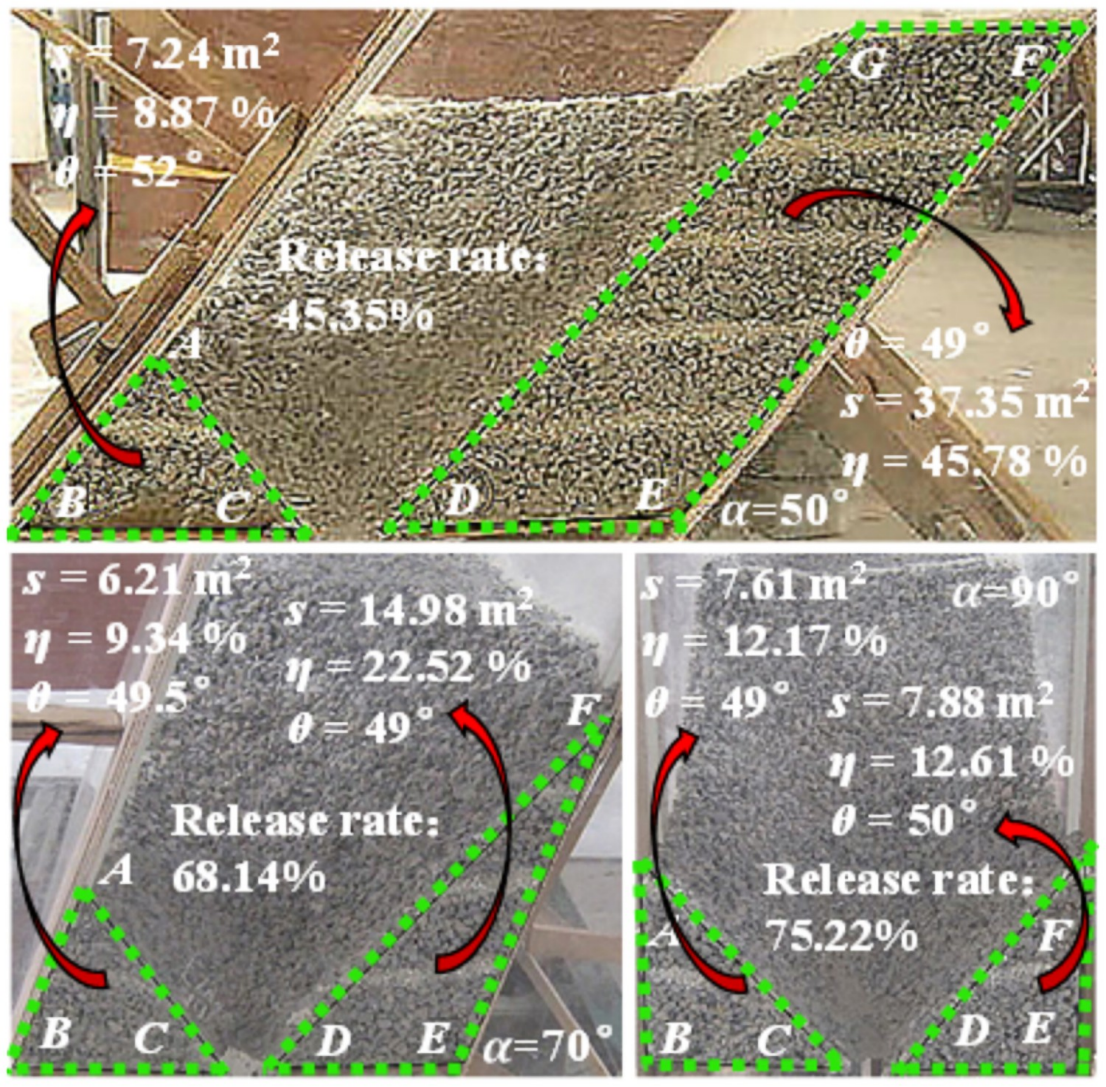
Resting forms at different inclinations.

In order to understand the influence of different coal seam inclination angles on coal body rest patterns, simulated coal caving experiments were conducted for different coal seam inclination models (α = 50°, 70°, 90°), as shown in Fig 4. After the coal body rests, the dip angle of the sliding surface is within the range of 49°-52°, so it can be seen that the dip angle of the sliding surface is equal to the natural resting angle. Statistical analysis shows that the greater the inclination of the coal seam, the greater the release rate of the coal body behind the tailstock, indicating that the tailstock is more viable.

#### On the dip profile

**Fig 5:**
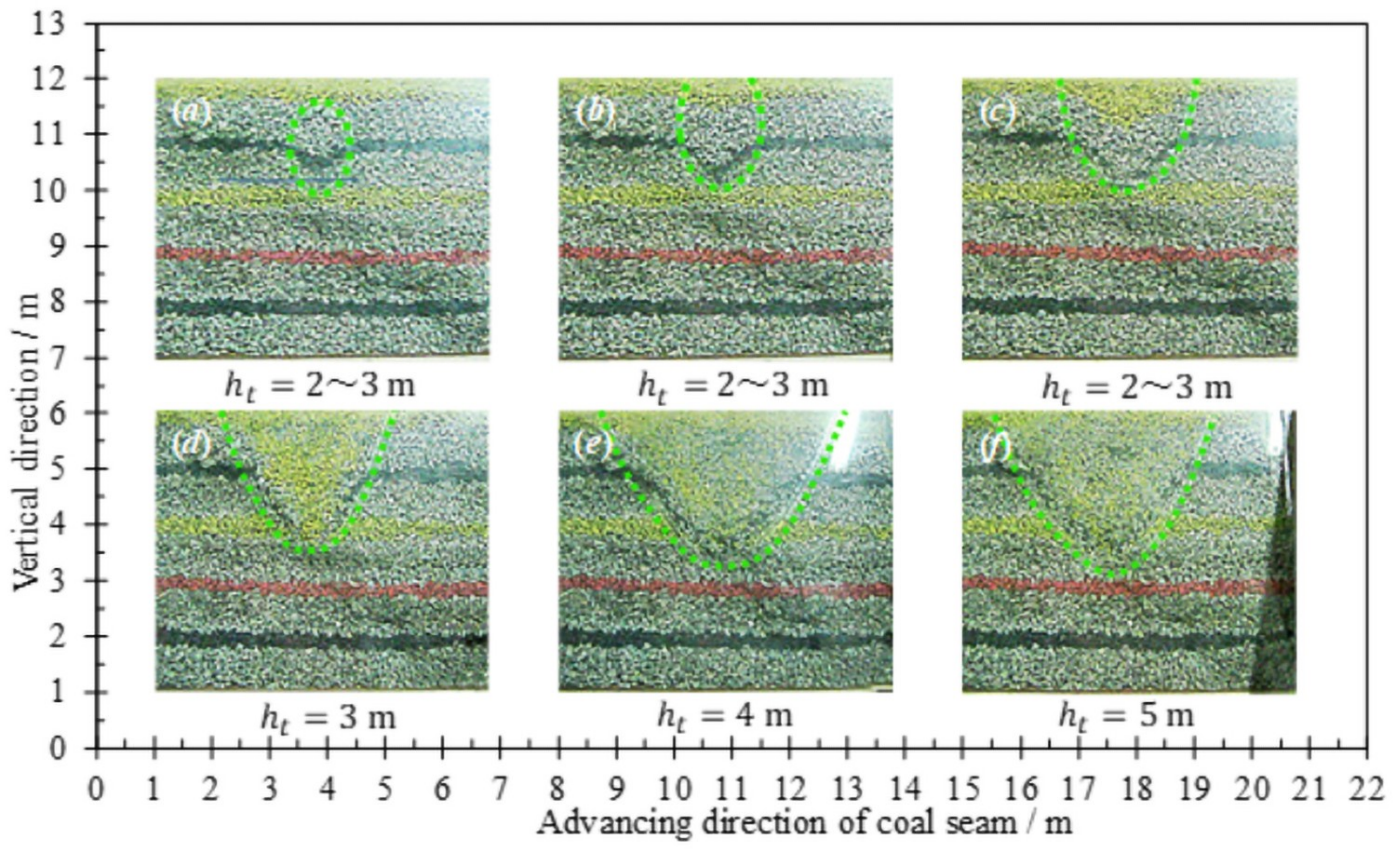
Loose form and discharging process of coal (on the dip profile).

At the coal seam level, the loose form and release process of the coal body are shown in Fig 5. When 0≤*h*_t_≤2.0 m, there is no substantial loosening of the coal body at the boundary of the roof, and the loosening range has not been extended to the roof. When 2.0 m≤*h*_t_≤3.0 m, the coal body above the yellow marker layer starts to loosen in the form of ellipsoid, and the loosening range extends to the boundary of the roof, as shown in (a) and (b). After the loosening range is extended to the top of the model, the upper coal body, like the lower coal body, is also extended along the direction of strike in the form of incomplete ellipsoid, as shown in Figs (c)-(f). It is not difficult to find that the maximum height of the releasing ellipsoid is basically the same as the height of the initial loosening of the coal body from the roof, which indicates that the support installation and model laying are more accurate.

### Caving rule of working face

#### On the strike profile

**Fig 6:**
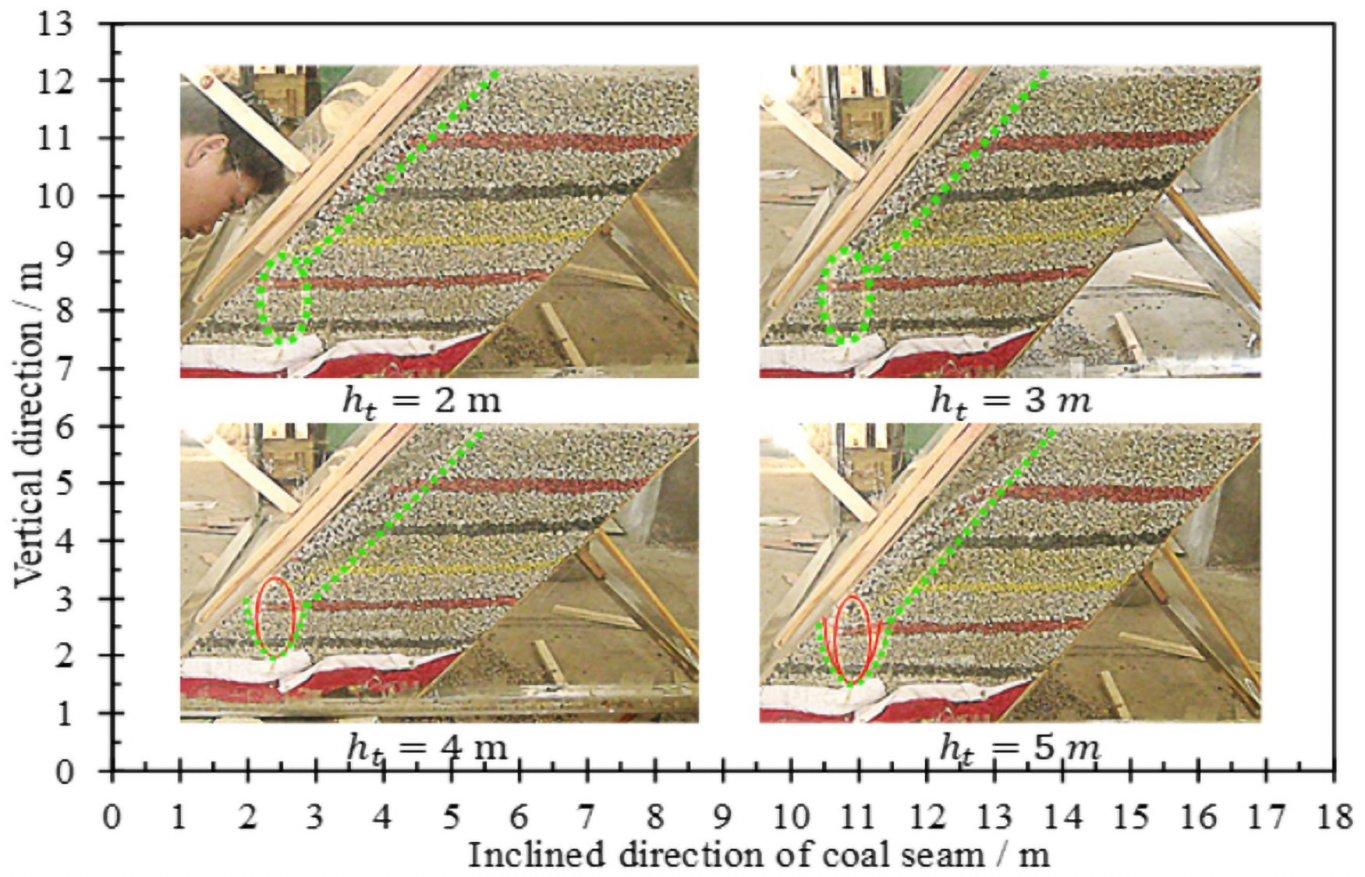
Loose form and discharging process of coal (on the strike profile).

As shown in Fig 6, the distance from the coal discharge port on the glass plate is 5.0 cm, and the early expansion process of the loosening body cannot be observed. However, according to the loosening and release of coal bodies on the strike profile, combined with the drawing law of coal bodies at the coal seam level (see below), it is not difficult to find that the shape of the release body and loose body under the coal seam is also an ellipsoid. When 2.0 m≤*h*_t_≤3.0 m, the loose ellipsoid has been extended to the roof, the upper coal body is loose along the roof boundary, and the expansion of loose ellipsoid shape shows significant hysteresis. When 4.0 m≤*h*_t_≤5.0 m, the broken ellipsoid in the lower coal body gradually expands, the loose range of the yellow marker layer gradually expands, the red marker layer begins to sink, and the loose range of the upper coal body gradually expands in the process of sliding towards the floor.

#### On the dip profile

**Fig 7:**
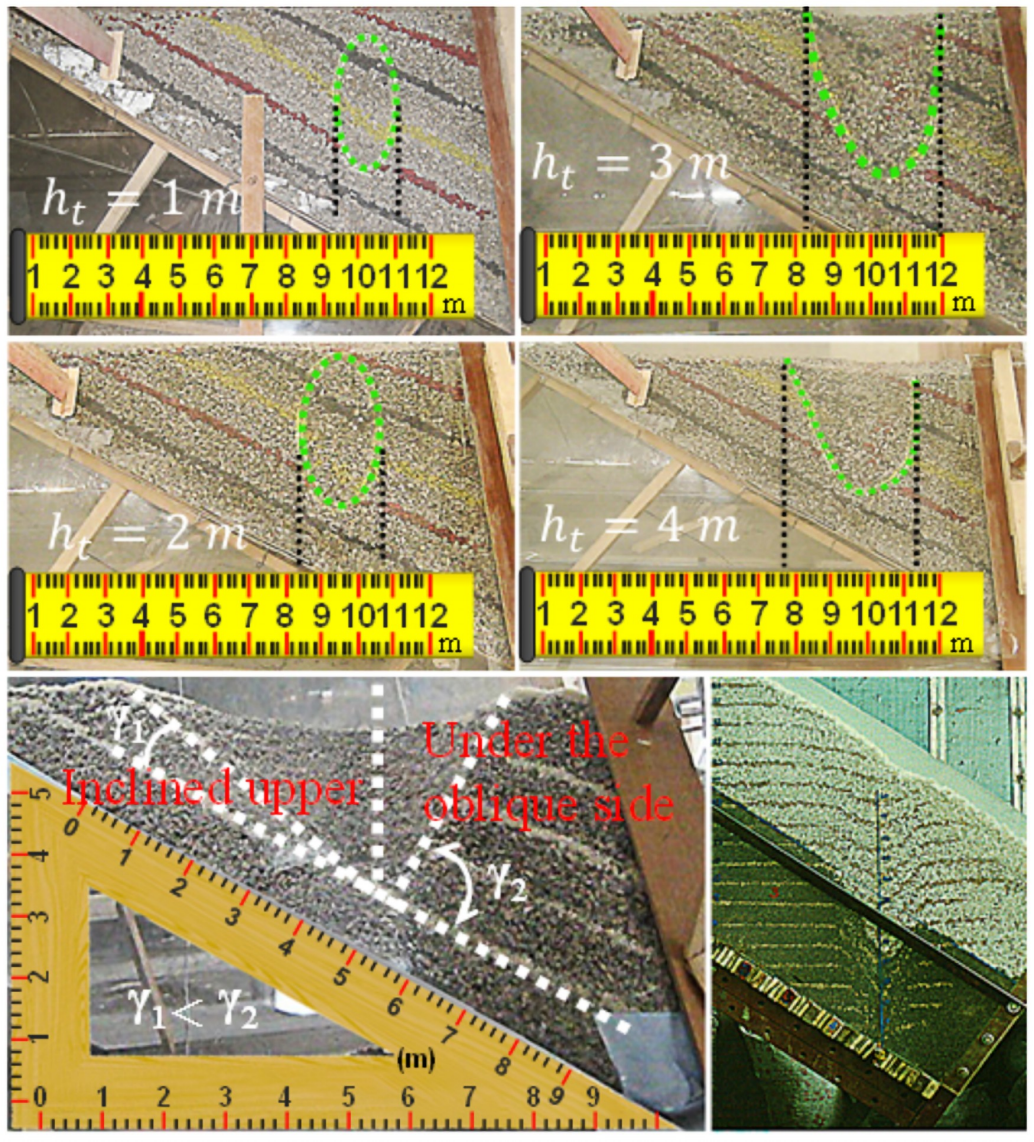
Loose form and discharging process of coal.

In the initial stage of coal caving, the caving body and loose body in the lower coal body expand in the form of ellipsoid, as shown in Fig 7. When the long axis of the loose ellipsoid is about 2.0 m, the red marker layer begins to loosen first. For the laying model, the vertical height from the coal chute to the roof is 2.3 m, which is consistent with each other, indicating that the support installation and model laying are more accurate. After the loosening range is extended to the roof, the upper coal body is also loosened by a new ellipsoid, when the setting out height is 1.0m, the long axis and short axis of the loose ellipsoid are 2.40 m and 1.11 m respectively, and when the setting out height is 2.0 m, the long axis and short axis are 2.88 m and 1.41 m respectively. It can be seen that the loose ellipsoid formed in the upper coal body have a smaller flatness, and the experimental constants *m* and *n* determined by the lower ellipsoid is no longer applicable. After the loosening range is extended to the top of the model, the upper coal body is loosened and released as a defective ellipsoid. Due to the pseudo-inclined arrangement of the working face, compared with the lower part of the central axis of the ellipsoid, the upper coal body is more easily released and the angle between the sliding surface and the floor is smaller.

### Different caving methods

**Fig 8:**
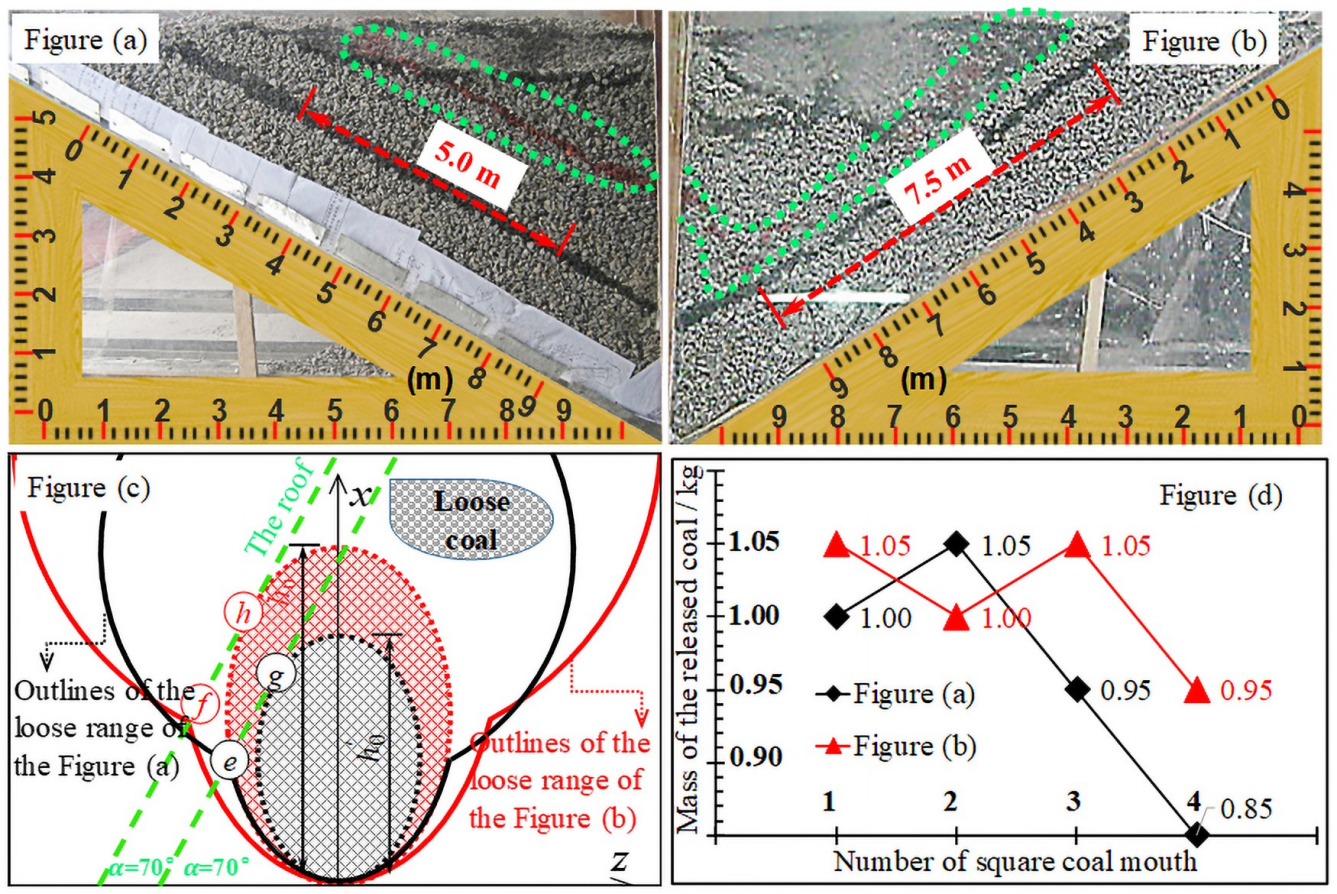
Caving form, loosening range and coal discharge.

In order to study the influence of the falling support mode on the coal body drawing law after the support, the coal releasing experiment is carried out for the coal seam with a dip angle of 70° and a thickness of 5.0 m, as shown in Fig 8.

Under the influence of the falling way of the support, the vertical distance between the coal discharge port and the roof is different from the lead sag distance, and the expansion degree of the loose ellipsoid is different before the loose range is extended to the roof. As a result, when the support falls down along the floor to discharge coal, the initial loosening position of the coal body on the roof is higher. Furthermore the height of the releasing ellipsoid, and the broken ellipsoid formed by the upper coal body is also larger. Similarly, looseness range at the top of the model, and the coal discharge quantity is also larger. It can be seen that, due to the influence of the boundary of the roof, the flexible shield support is more favorable for the caving of the coal behind when it falls along the floor.

## Numerical simulation

**Fig 9:**
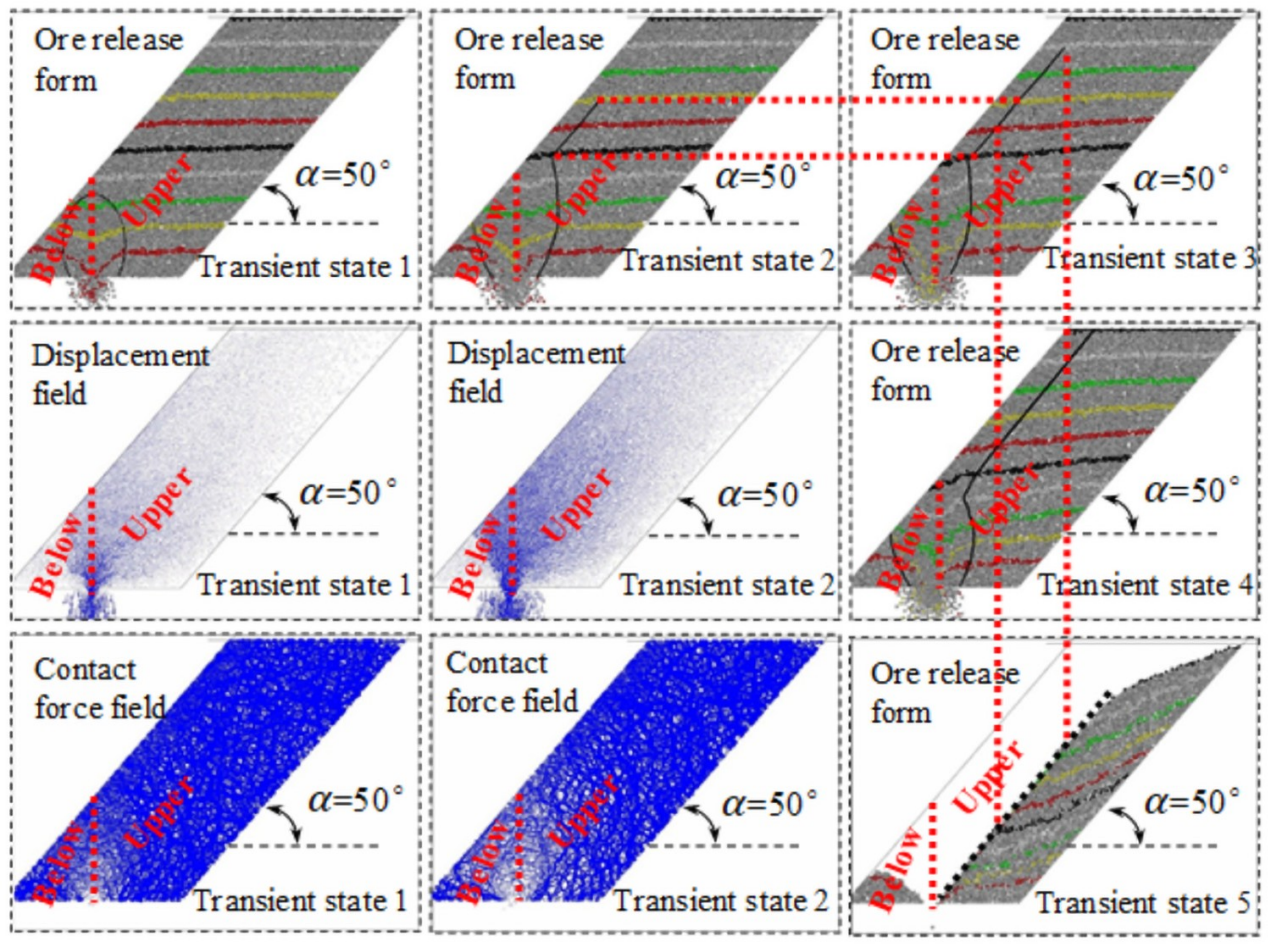
Falling forms, displacement field and contact force field of transportation roadway.

**Fig 10:**
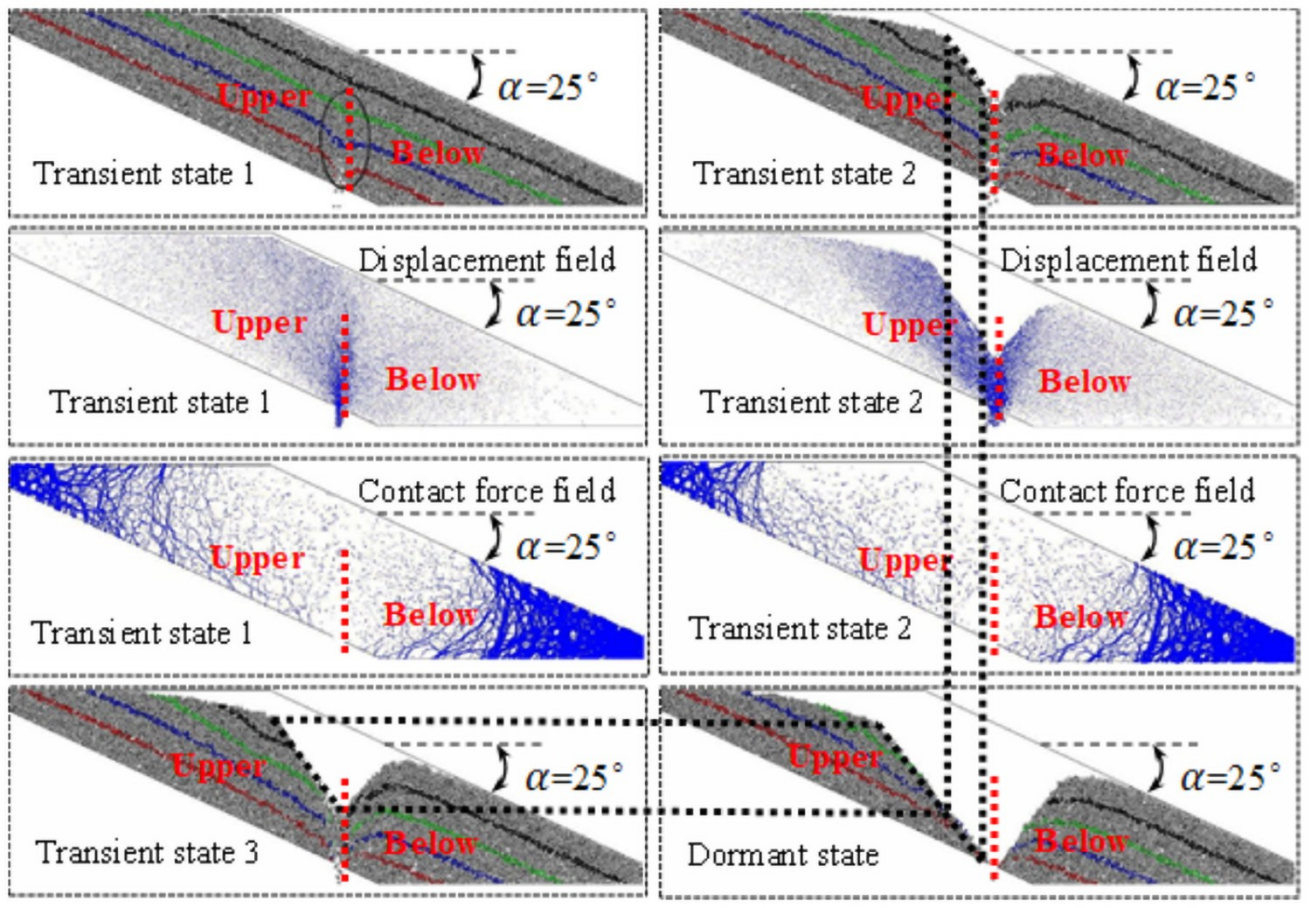
Falling form, displacement field and contact force field of transportation roadway.

In the numerical simulation, different colors are used to distinguish the coal body and the marking layer, and the particle size of the coal body follows the Gaussian distribution, ranging from 0 to 300 mm, the physical and mechanical parameters of the loose coal body behind the frame are listed in Table 1. The falling form, displacement field and the contact force field of coal behind the tailstock are shown in Fig 9. The falling form, displacement field and the contact force field of coal behind the working face support are shown in Fig 10.

The comparison between Figs 9 and 10 is consistent with the results of the physical simulation, no matter whether the tailstock or the working face support is used to discharge coal, the falling form, displacement field and contact field all show an obvious similarity. Before the loosening range is extended to the roof, both the releasing body and the loosening body expand in the form of an ellipsoid. After the loosening range is extended to the roof, the upper coal body is loosened by the sliding face to the floor translation, while the lower coal body is released due to the expansion of the incomplete ellipsoid. Consistent with the results of physical simulation, the coal bodies on both sides are stable at the back of the support in the form of natural rest, but it is easier to release the coal body above the slope, while the lower coal body is released due to the expansion of the broken ellipsoid. During this period, the displacement of the coal body at the top of the slope increases significantly, while the displacement of the coal body at the floor of the slope is still small, the loosening range, displacement field and contact force field of the coal bodies at both sides show obvious differences, and the included angle between the slip surface and the floor is also smaller.

Displacement field: in the early stage of coal discharge, the displacement near the coal discharge port is the largest and the falling speed is the fastest. The displacement and velocity of the coal body below the roof are the next, and the displacement and velocity of the coal body at the floor (near the coal discharge outlet) are the smallest. Moreover, from the roof to the roof direction, the displacement gradually weakens, and the continuous release will cause the displacement of the coal body to gradually increase, the depth of the coal mining operation affects the area gradually expands to the floor, which is consistent with the expansion trend of the coal body loosening range.

Contact force field: in the early stage of coal discharge, an ellipsoid area with small contact force was formed near the coal discharge port, and the contact force near the central axis of the ellipsoid was generally smaller. By comparing the distribution characteristics of instantaneous displacement field and contact force field of coal discharge, it can be found that the contact force is usually small in the area with large displacement, and the corresponding relationship is almost synchronous in the whole process of coal discharge. Based on this cognition, in the process of continuous coal discharge, the looseness range and displacement of the coal body gradually increase, and the ellipsoid area with small contact force gradually expands, indicating that the arch formation probability of the coal body behind the support is gradually reduced.

## Analysis and discussion

### General rules of coal caving

**Fig 11:**
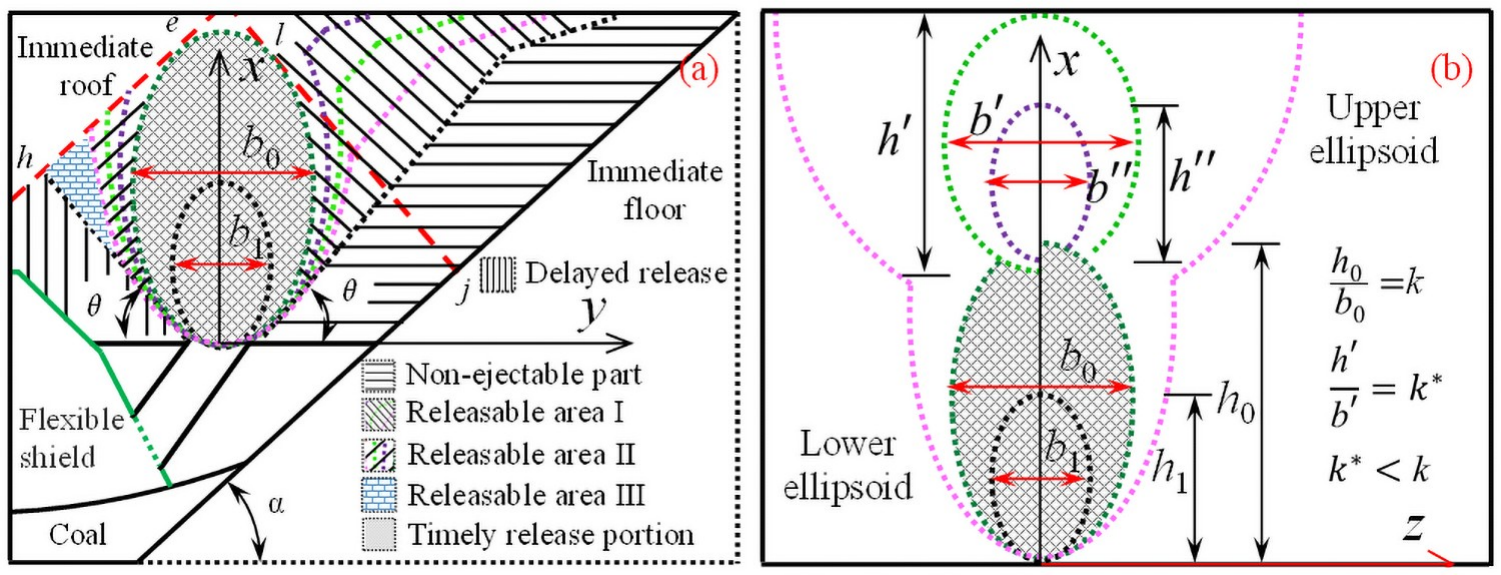
Release process and loose form of the coal body. (a) On the strike profile. (b) On the dip profile.

Physical simulation and numerical simulation show that the shape of the released body and the loose body are ellipsoid, and the displacement field, contact force field and natural rest form are significantly similar. Based on the drawing theory of ellipsoid, the release process and loose form of the coal body are shown in Fig 11.

Based on the time effect and regional difference of the drawing process, the drawing of coal body behind the support can be divided into four zones. The first zone is the timely release portion. The second zone is the releasable area, including releasable area I, releasable area II, and releasable area III, as shown in Fig (a). After the ellipsoid is extended to the roof, these coal bodies can be released through continuous coal caving operation on the premise of dilution. The third zone is the delayed release portion, which will be released in the fall of the support after the termination of the release, or will be released from the next round of coal caving process. The fourth zone is the non-discharging part, but after optimizing the coal discharging process parameters, such as the support structure form, the discharging height and the mining-releasing ratio, the coal body still has the possibility of releasing, thereby reducing the coal loss behind the support and improving the working face recovery rate.

On the strike profile: affected by many boundary conditions, such as roof, floor, front gangue, top gangue, support structure, etc., the lower coal body will be discharged with the expansion of the discharging range, or it will naturally rest behind the support. The upper coal body is loosened in such a way that the sliding surface moves parallel to the floor. The discharge of the coal bodies above the diagonal is easier, and the angle between the slip surface and the floor is also smaller.

On the dip profile: the release of coal body can be regarded as the release of loose particles without boundary conditions, and the single ore drawing volume is mainly affected by the coal drawing distance and coal drawing step distance. Both the upper and lower coal bodies are released in the form of ellipsoid or incomplete ellipsoid, with the difference that the oblateness of the upper ellipsoid is smaller. Therefore, the experimental constants m and n determined by the morphology of the lower ellipsoid are no longer applicable.

### Parameters optimization

After the loosening range is extended to the gangue boundary, the continuous coal discharge will face the challenge of ore depletion. It is of great significance to discuss the drawing law of coal body behind the flexible shield support for optimizing the coal discharges process parameters and reducing coal loss [32, 33]. Based on the physical simulation and numerical simulation, whether tailstock or face support, the shape and boundary conditions of the caving body are basically the same when caving along the roof; while the shape and boundary conditions of the caving body are different when tailstock and face support are caving along the floor. The shape, boundary conditions and process parameters of the drawing body are listed in Table 3.

**Table 3:** Shape, boundary conditions and process parameters of the drawing body.

Fall Along the Roof	Fall Along the Floor		Distance of the Coal Outlet	
*l*_*oc*_ = *l*_*hn*_, *l*_*oc*_ = *l*_*sn*_	Haulage roadway	Working face	Haulage roadway	Working face
Eqs (1–1) and (1–2)	Eqs (2–1) and (2–2)	Eqs (3–1) and (3–2)	Eqs (4–1) and (4–2)	Eqs (5–1) and (5–2)
Eqs (1–3)	Eqs (2–3)	Eqs (3–3)	Eqs (4–3)	Eqs (5–3)
Eqs (1–4)	Eqs (2–4)	Eqs (3–4)	Eqs (4–4)	Eqs (5–4)
Eqs (1–5)	Eqs (2–5)	Eqs (3–5)	/	/

As mentioned above, the formulas in Table 3 are listed as follows:

$$\begin{array}{lll} \{\begin{array}{ll} y^{2}=mh^{-n}x(h-x) & \left(1-1\right)\\ x=\left(y+l_{oc}\right)\text{tan}\;\alpha & \left(1-2\right)\\ h_{0}=\frac{2l_{\text{oc}}\left(\sqrt{1+m\;{\text{tan}}^{2}\alpha }-1\right)}{m\;\text{tan}\;\alpha } & \left(1-3\right)\\ M_{f}=\frac{2\sqrt{1+m\;{\text{tan}}^{2}\;\alpha }}{\sqrt{1+m\;{\text{tan}}^{2}\;\alpha }+1}l_{oc} & \left(1-4\right)\\ k_{cf}=\frac{\left(M-2l_{oc}\right)\sqrt{1+m\;{\text{tan}}^{2}\;\alpha }+M}{2l_{oc}\sqrt{1+m\;{\text{tan}}^{2}\;\alpha }} & \left(1-5\right) \end{array} & \{\begin{array}{ll} \zeta =a_{1}{\left(\;\frac{\varphi }{L}\right)}^{b_{1}} & \left(2-1\right)\\ M_{f}\;=2\zeta & \left(2-2\right)\\ h_{0}=\frac{\varphi }{\text{cos}\;\;\alpha }{\left(\frac{90{}^{\circ}-\alpha }{a_{1}}\right)}^{-b_{1}} & \left(2-3\right)\\ M_{f}=\sqrt{mh_{0}^{2-n}} & \left(2-4\right)\\ k_{cf}=\frac{M-\sqrt{mh_{0}^{2-n}}}{\sqrt{mh_{0}^{2-n}}} & \left(2-5\right) \end{array} & \{\begin{array}{ll} y^{2}=mh^{-n}x\left(h-x\right) & \left(3-1\right)\\ x=\text{tan}\;\theta \left(l_{op}-y\right) & \left(3-2\right)\\ h_{0}=\frac{2l_{op}}{m\;\text{tan}\;\theta }\left(\sqrt{1+m\;{\text{tan}}^{2}\;\theta }-1\right) & \left(3-3\right)\\ M_{f}=l_{op}\left[\frac{\text{tan}\;\alpha }{\text{tan}\;\theta }\frac{\left(\sqrt{1+m\;{\text{tan}}^{2}\;\theta }-1\right)}{\left(\sqrt{1+m\;{\text{tan}}^{2}\;\theta }-1\right)}+1\right]\text{sin}\;\alpha & \left(3-4\right)\\ k_{cf}=\frac{M\;\text{sin}\;\theta \;\text{cot}\;\alpha \left(\sqrt{1+m\;{\text{tan}}^{2}\;\alpha }-1\right)}{l_{op}\text{sin}\left(\alpha +\theta \right)\left(\sqrt{1+m\;{\text{tan}}^{2}\;\theta }-1\right)} & \left(3-5\right) \end{array} \end{array}$$

$$\begin{array}{ll} \{\begin{array}{ll} y^{2}=mh^{-n}x\left(h-x\right) & \left(4-1\right)\\ h=2x & \left(4-2\right)\\ \text{s}=\sqrt{mh^{2-n}} & \left(4-3\right)\\ \text{d}=\sqrt{mh^{2-n}} & \left(4-4\right) \end{array} & \{\begin{array}{ll} y^{2}=mh^{-n}x\left(h-x\right) & \left(5-1\right)\\ y=-\text{cot}\;\beta \left(x-\frac{h}{2}\right) & \left(5-2\right)\\ \text{s}=\sqrt{\frac{mh^{2-n}\left(1+{\text{cot}}^{2}\;\beta \right)}{{\text{cot}}^{2}\;\beta +mh^{-n}}} & \left(5-3\right)\\ \text{d}=\sqrt{\frac{mh^{2-n}\left(1+{\text{cot}}^{2}\;\beta \right)}{{\text{cot}}^{2}\;\beta +mh^{-n}}} & \left(5-4\right) \end{array} \end{array}$$

The flexible shield support falls down along the roof: when the support of the working face releases coal, the roof (straight *eh*) and the floor (straight *mn*) are the main factors that affect the law of coal release, at this time, *l*_*oc*_ = *l*_*hn*_. The reasonable coal discharge thickness should meet the requirement that when the height of the ellipsoid is maximized, it is tangent to the roof and floor. When discharging coal from tail support, the front gangue (straight rs) and the floor are the main factors affecting the law of coal discharge, and then *l*_*oc*_ = *l*_*sn*_. The reasonable thickness of coal discharge should meet the requirements, when the height of ellipsoid is maximized, it is tangent to the front gangue and floor at the same time. For a specific coal seam, the inclination angle and thickness M are constants. After the vertical distance from the coal discharge orifice to the roof and the plumb distance is determined, the shape of the ellipsoid and the reasonable recovery and release ratio is basically determined.

The tailstock falls along the floor: when the height of the released ellipsoid is greater than the vertical distance due to the influence of the top and floor, the released ellipsoid will deflect [21]. According to the results of experimental statistics, when the x-axis is parallel to the coal seam roof, the developed ellipsoid is the best, and the released rate of the coal body is the largest. When the thickness of the coal seam is equal to twice the eccentricity, the shape of drawing ellipsoid and reasonable mining-drawing ratio is basically determined.


$$\frac{h_{1}}{\text{tan}\;\alpha }=\frac{\varphi }{\text{cos}\;\alpha }{\left(\frac{90{}^{\circ}-\alpha }{a_{1}}\right)}^{-b_{1}}$$

The support of working face falls along the floor: The top gangue boundary (straight *ep*) and the top plate (straight *eh*) are the main factors affecting the coal drawing law. The reasonable coal discharge thickness should meet the requirements, when the height of the ellipsoid is maximized, it is tangent to the top gangue and the roof, and the inclination direction of the straight ep is related to the natural resting form of the coal (*k* = tanθ). Therefore, for a specific coal seam, the reasonable height of the release and the ratio of production to release are basically determined.

Reasonable coal caving distance: As shown in Table 3, *s* is the axial distance when two ellipsoids are tangent, and *d* is the coal caving distance. Because of the limitation of the flexible shield support, the coal should be discharged from the lower end to the upper end and from the single opening. When *d* = *s*, the height of the adjacent ellipsoids is the same and tangent to each other; when *d* > *s*, the height of the adjacent ellipsoids is the same, but there will be a lot of back coal that cannot be released; when *d* < *s*, the height of the second coal outlet will be reduced, and the third coal outlet will be larger than the second coal outlet, and so on, which will cause a lot of loss of back coal. Therefore, when *d* = *s*, the release rate of coal body behind the support is the largest, the loss of coal is the smallest, and the recovery rate of working face is higher.

## Industrial experiment

### Engineering situation

The elevation of the coal seam to be mined in Bai-Ji Mine is -800 m to -500 m, the seam inclination angle is 50°, the thickness is 5.0 m, the coal quality is relatively soft, and it is easy to adopt the coal caving process, the supports are used to drop coal along the floor. In view of the transport capacity of the scraper conveyor, the working face mining ratio is determined to be 3:7. With the method of uphill layout, the return air roadway and transportation roadway are arranged along the strike, the working face width is 90 m, the continuous advance length is 500 m, the pseudo-dip angle is 25°, and the vertical height of the section is 45 m. The cutting hole, return airway and travelling roadway are drift along the floor, all of which are supported by the articulated roof beam.

**Fig 12:**
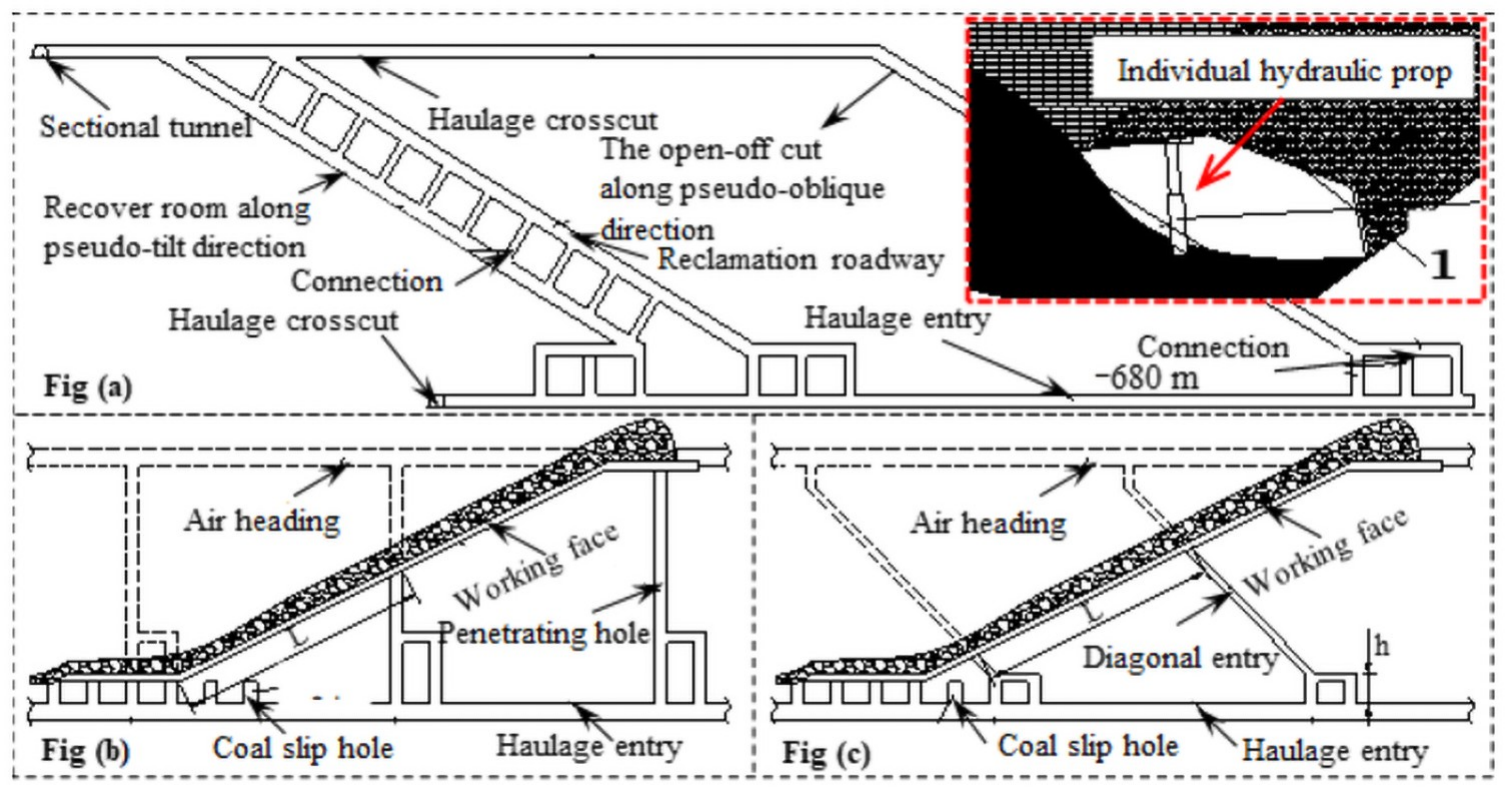
The schematic diagram of the working face layout. Fig (a). The open-off cut and recover rooms are all arranged along the pseudo-oblique direction. Fig (b). The coal-sliding hole was arranged in the working face. Fig (c). The pseudo-inclined roadway was arranged in the working face.

In order to realize the plan of sublevel caving, a reclamation roadway and a connection were arranged in the working face, which was divided into several areas with a length of 30–40 m. This not only alleviates the difficulty of transporting materials caused by the height difference, accelerates the tunneling speed, but also simplifies the installation, adjustment and recovery procedures of supports, and optimizes the ventilation system. Moreover, in order to adjust the falling height of the support, an individual hydraulic prop was erected under the flexible shield support. The schematic diagram of working face layout is shown in Fig 12.

### Parameters of caving

1. Parameters of coal caving of flexible shield support in the tail caving section of transportation gateway
  According to the analysis in Table 3, the deflection of the released ellipsoid occurs. Based on the experimental results of similar material simulation and numerical simulation, when the experimental constant *m* is 0.78, *n* is 1.32, and the thickness of the drawing coal *M*_*f*_ is 3.5 m, the optimal length axis *h*_0_ of the released ellipsoid development is:
$$h_{0}=\sqrt[{2-n}]{\frac{M_{f}^{\;2}}{m}}=6.51\;\text{m}$$
  According to the length, axis of the ellipsoid is 6.51 m, the reasonable coal discharge distance can be obtained as follows:
$$d=\sqrt{0.78\times 6.51^{2-1.32}}=1.67\;\text{m}$$
2. Coal discharge parameters of flexible shield support in pseudo-inclined working face
  According to the analysis in Table 3, when the discharging thickness *M*_*f*_ is 3.5 m, *l*_*op*_ is 2.29 m, and the reason discharging height is:
$$h_{0}=\frac{2l_{op}}{m\text{tan}\theta }\left(\sqrt{1+m{\text{tan}}^{2}\;\theta }-1\right)=2.23\;\text{m}$$
  According to the height of coal discharge of 2.23 m, the reasonable distance of coal discharge can be obtained as follows:
$$d=\sqrt{\frac{0.78\times 2.23^{2-1.32}\times \left(1+{\text{tan}}^{2}\;25{}^{\circ}\right)}{{\text{tan}}^{2}\;25{}^{\circ}+0.78\times 2.23^{-1.32}}}=1.40\;\text{m}$$

### Experimental verification

#### Physical simulation

**Fig 13:**
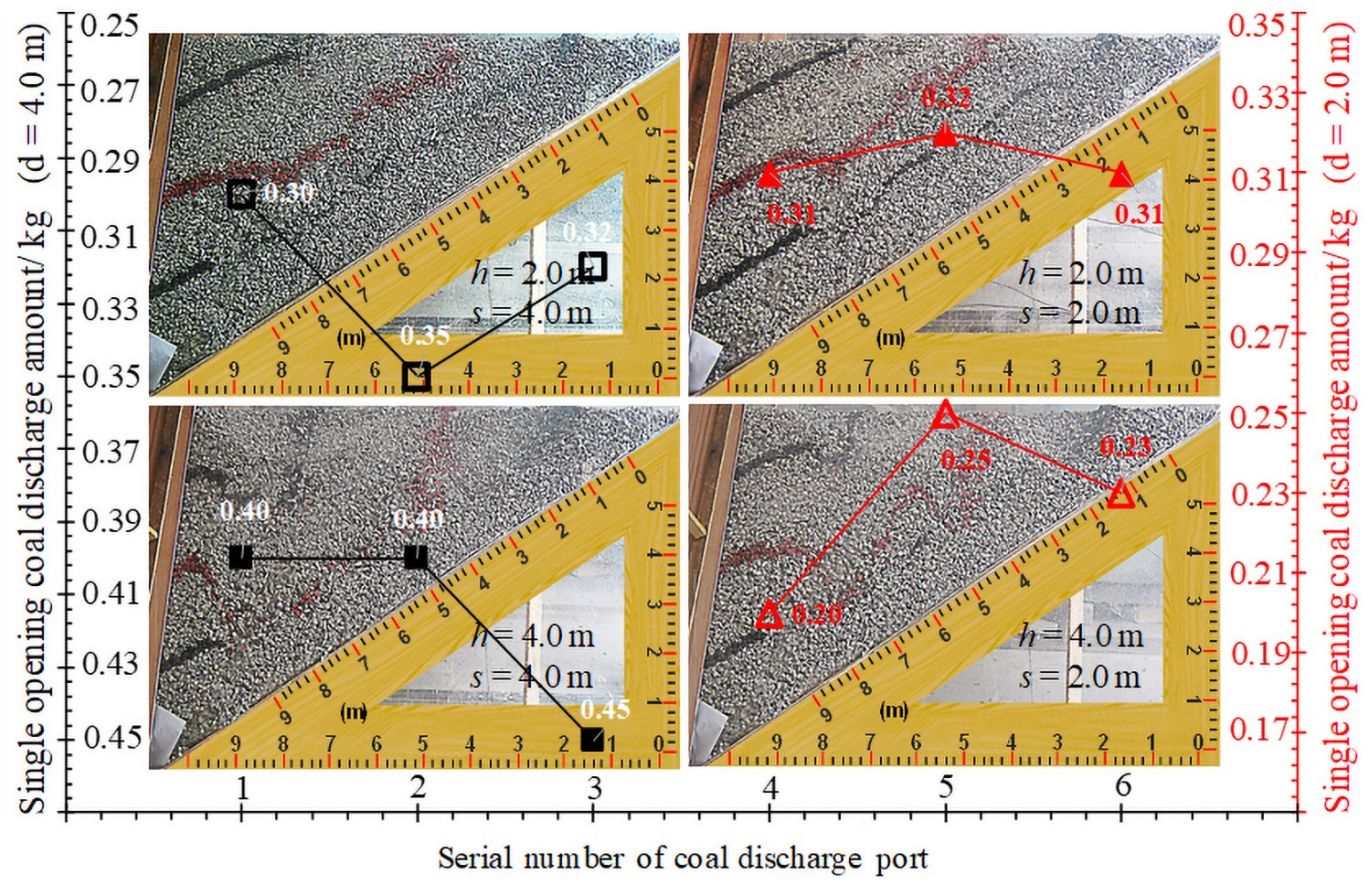
Release form and coal release amount of media.

In order to check the rationality of the height and interval of coal discharge, we carried out simulation experiments of similar materials. In the process of coal discharge, the single-wheel sequential single-port coal discharge is adopted. In the case of different height and interval of coal discharge, the falling form of coal behind the flexible shield support and the single-port coal discharge amount is shown in Fig 13.

The height of the coal discharge is 2.0 m: when the coal discharge interval is 4.0 m, the coal discharge amount is 0.97 kg, and when the coal discharge interval is 2.0 m, the coal discharge amount is 0.94 kg, which is the same as that of the two. The height of coal discharge is 4.0 m: when the coal discharge interval is 4.0 m, the coal discharge amount is 1.25 kg, and when the coal discharge interval is 2.0 m, the coal discharge amount is 0.68 kg, which is far from the coal discharge amount when the coal discharge interval is 4.0 m, accounting for about 54.4%. Moreover, in the coal layer, the degree of distortion of the marker layer is also different. When the interval of coal discharge is 4.0 m, the degree of distortion of the marker layer is relatively large, indicating that there is a large amount of back-coal loss; when the coal discharge interval is 2.0 m, the distortion degree of the marker layer is small and the subsidence is uniform, indicating that the amount of back-coal loss is small. Based on the above analysis, when the coal discharge height is 2.0 m, the coal discharge interval should be less than 2.0 m. When the coal discharge height is 4.0 m, the coal discharge interval should be between 2.0–4.0 m. Therefore, for the Bai-Ji Mine, when the height of the coal put in the working face is 2.23 m, the coal discharge interval of 1.40 m is reasonable.

#### Numerical simulation

**Fig 14:**
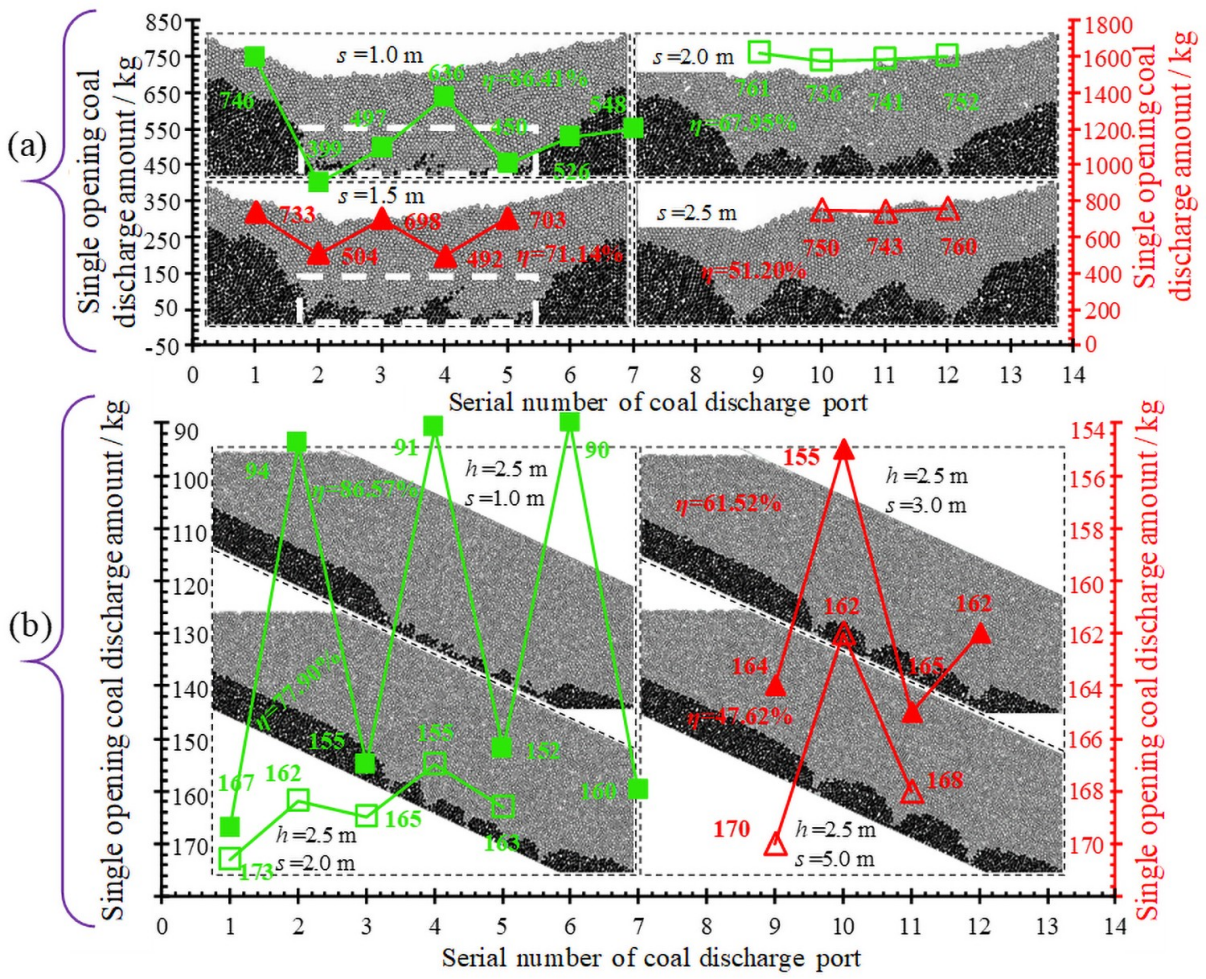
Stacking patterns of media at different coal caving distances. As shown in Fig (a), when the coal discharging interval is 1.0 m and 1.5 m, the fluctuation ranges of the coal discharging quantity of a single port is large, and the backside coal is piled into an inclined strip area, when the coal discharging interval is 1.5 m, the backside coal dispersion degree is smaller and the accumulation area is larger. When coal discharge distance is greater than 2.0 m, the fluctuation range of the coal discharge quantity of a single port is small, and there is almost no discrete state of back-coal, but the accumulation area is positively correlated with the coal discharge distance. For Bai-Ji mine, when the tailstock caving height is 6.51 m, the caving interval is 1.67 m. As shown in Fig (b), when the coal caving interval is 1.0 m, the fluctuation range of single coal caving is large, and the back coal is relatively discrete. When the distance of coal caving is 2.0 m, 3.0 m, and 5.0 m, the fluctuation range of single coal caving is small, and there is almost no discrete state of back coal. Consistent with the tailstock caving, the stacking area of back coal is positively correlated with the caving distance. For Bai-Ji Coal Mine, when the caving height of the working face is 2.23 m, the drawing interval of coal is more reasonable at 1.40 m.

Through numerical simulation, the distance between two positions (working face and haulage roadway) is checked. When building the model, the height of tailstock caving is 6.5 m, and the height of working face support caving is 2.5 m. During the simulation of coal caving, the principle of gangue parking is followed, the stacking shape and coal caving amount of the coal body behind the support with different caving intervals are shown in Fig 14.

The results of numerical simulation show that too small coal discharge interval will become one of the main factors affecting the single-port coal discharge amount, and vice versa, having little effect on the single-port coal discharge amount. With the increase of the coal separation distance, the loss of ridge coal will gradually increase, and the rate of coal discharge after the support will gradually decrease.

### Experimental results

Compared with the early test of slicing blasting in the working face (Scheme A), many difficulties have been avoided in the mining of caving coal behind the flexible shield support (Scheme B). For example, laying false roof, roof caving, high tunneling rate, serious material consumption, poor production conditions, high labor intensity, low recovery rate and poor economic benefits, etc. During the mining period, due to the accumulated conventional experience of scheme A, no major technical problems appeared. The output of the working face was about 1580 t/d, and the recovery rate was as high as 85.6% combined with the excavated coal output, which basically met mine production capacity (0.50 Mt/a).

As listed in Tables 4 and 5, compared with the Scheme A (2.35 t), the Scheme B (6.33 t) not only improves everyone’s work efficiency, but also has obvious advantages in equipment allocation, material consumption and labor organization. According to the cost per ton of coal, the depreciation expense of equipment is 2.21 yuan and 0.65 yuan respectively, the electricity cost is 0 yuan and 0.39 yuan respectively, the labor cost is 42.28 yuan and 18.46 yuan respectively, the material cost is 2.75 yuan and 2.16 yuan respectively, the roadway maintenance cost is 16.12 yuan and 9.31 yuan respectively, and the working face migration cost is 0.35 yuan and 0.20 yuan respectively. Accumulated production costs are 64.71 yuan and 33.17 yuan respectively.

**Table 4:** Equipment and materials in the working face.

Scheme A			Scheme B		
Equipment and materials	Quantum	Amount	Equipment and materials	Quantum	Amount
Individual hydraulic prop	1540	123.20	Individual hydraulic prop	180	14.40
Articulated roof beam	1320	79.20	Articulated roof beam	40	2.40
Steel rope	594	0.48	Steel rope	880	1.58
Liquid injection gun	12	0.24	Liquid injection gun	6	0.12
Emulsion pump	2	200	Emulsion pump	1	100
Iron Palm	1067	0.53	I Beam 11^#^	7161	154
Round timber	117	0.35	U clamp, Clamping plate	7700	46.74
Pressure pipe	560	0.11	Wire netting	660	1.16
Spillplate	500	0.50	Nut	15400	0.6
Summation	/	404.61	Summation	/	321.00

**Table 5:** Personnel organization and distribution in the working face.

Scheme A				Scheme B			
Activity requirement	Morning	Midday	Evening	Activity requirement	Morning	Midday	Evening
Drilling, Blasting	4	4	/	Drilling, Blasting	8	8	8
Monitor, Assist	3	2	/	Monitor, Assist	3	3	3
Stock	2	2	/	Stock	4	4	4
Maintain	/	/	6	Maintain	1	1	1
Grain	1	1	/	Forepoling, Installation	4	4	4
Complex operation	39	39	/	Undercarriage	6	6	6
Watch keeper	7	7	/	Planing, Sneak coal	4	4	4
Support crew	4	1	/	Support crew, Drawing	16	16	16
Summation	60	6	6	Summation	46	46	46

The Scheme B not only achieved ideal economic benefits, but also avoided the capital investment of 30–50 million yuan (plan to upgrade the equipment of the working face after the failure of the Scheme A), this is undoubtedly an economical and reliable attempt for the aging mine with exhausted resources, which innovated the coal mining technology and extended the mine service life. The successful practice shows that, ellipsoidal ore drawing theory has certain rationality in the optimization of coal drawing parameters behind flexible shield support in pseudo-inclined working face of steep coal seam, at the same time, it also provides a successful model for similar mining.

## Conclusions

Through physical simulation, numerical simulation, theoretical analysis and other research methods, this paper systematically analyzes the coal release law behind the flexible shield support in the pseudo-inclined working face of steep coal seam. Combined with complex boundary conditions, on the basis of establishing internal relations among various parameters, a concrete method of optimizing coal caving parameters was put forward, and the industrial test was carried out. The research results show that:

1. The complex boundary condition is the main reason that affects the coal release rule behind the flexible shield support in the pseudo-inclined working face of steep inclined coal seam. Before reaching the roof, both the released body and loose body expand in the form of ellipsoid; After reaching the roof, both the releasing body and the loose body form an incomplete ellipsoid butted up and down. Compared with the lower ellipsoid, the oblateness of the upper ellipsoid is smaller, and the constants m and n determined by experiments are also different.
2. Along the dip direction, the coal emission can be equivalent to an ideal loose body without boundary conditions; Along the strike direction, there are obvious regional differences in coal discharge on both sides of the long axis, which can be roughly divided into four parts: timely discharge, delayed discharge and non-discharge, among which the delayed discharge coal will be discharged in the next round of coal discharge. In addition, in two directions, when the releasing body and loose body reach the roof, there is a short delay in the expansion speed.
3. Whether it is the support of the working face or the tailstock at both ends, falling the support along the floor is more conducive to the release of coal, and the initial loose position of coal at the roof is lower, and the release height, loose range and release rate are all larger. There is an obvious correlation among coal caving shape, displacement field and the contact force field, and there are obvious differences between the loosening range, displacement field and the contact force field on both sides of the long axis. The larger the displacement, the smaller the contact force, and this correlation is valid for the whole coal caving process.
4. Under the complex multilateral boundary conditions, the internal relations of coal caving parameters are established. The successful test shows that the ellipsoidal ore drawing theory is reasonable for the analysis of the coal discharge rule behind the flexible shield support and the optimization of parameters in the pseudo-dip angle working face of steep coal seam.

## Supporting information

S1 File (ZIP)

## Abbreviations

*a*_1_: Specific experimental constants
*b*_1_: Specific experimental constants
*c*: Coefficient of loose range
CGDF: Coal-gangue dividing face
*d*: Spacing of the coal discharge port along the pseudo-inclined direction
*h*: Height of the released coal
*h*_0_: Reasonable height of the released coal
IEZ: Isolated Extraction Zone
IMZ: Isolated Movement Zone
*M*: Coal seam thickness
*m*: Velocity distribution index
*M_f_*: Reasonable thickness of coal to be released
*n*: Index of moving trace of ellipsoid
*s*: Distance between the axes of the IEZ
*x*: Vertical coordinates of a cylindrical coordinate system
*α*: Dip angle of coal seam
*β*: Pseudo-inclination of the working face
*θ*: Natural angle of repose
