# Supplementary material for: Law of coal caving behind the flexible shield support in pseudo-inclined working face
Source: PLoS One. 2021 Dec 30;16(12):e0261355. doi: 10.1371/journal.pone.0261355 (PMC8717996; doi:10.1371/journal.pone.0261355)
Supplement: S1 File — (ZIP) [file pone.0261355.s001.zip › Supporting information/S1 Table.docx]

**Table 1. Physical parameters of coal and gangue.**

| Rock mass | Density / kg·m^-3^ | Normal stiffness / kN·m^-1^ | Shear stiffness / kN·m^-1^ | Frictional coefficient | Repose angle / ° | Cohesive force / N |
| --- | --- | --- | --- | --- | --- | --- |
| Coal | 1400 | 2.0×10^5^ | 2.0×10^5^ | 0.40 | 50 | 0 |
| Gangue | 2650 | 4.0×10^5^ | 4.0×10^5^ | 0.40 | 50 | 0 |
