# Supplementary material for: Law of coal caving behind the flexible shield support in pseudo-inclined working face
Source: PLoS One. 2021 Dec 30;16(12):e0261355. doi: 10.1371/journal.pone.0261355 (PMC8717996; doi:10.1371/journal.pone.0261355)
Supplement: S1 File — (ZIP) [file pone.0261355.s001.zip › Supporting information/S2 Table.docx]

**Table 2. Projection area and discharge rate of each part of coal body.**

| Release height /m | Triangle *abc* | | Polygons *edcfg* | | Outgoing triangle | |
| --- | --- | --- | --- | --- | --- | --- |
|  | Surface / m^2^ | Percent / % | Surface / m^2^ | Percent / % | Surface / m^2^ | Percent / % |
| 1 | 6.02 | 18.46 | 26.61 | 81.54 | 0 | 0 |
| 2 | 4.60 | 14.08 | 25.34 | 77.66 | 0 | 0 |
| 3 | 4.44 | 13.61 | 21.23 | 65.04 | 0.67 | 2.06 |
| 4 | 4.25 | 13.04 | 19.98 | 61.21 | 2.15 | 6.60 |
| 5 | 3.52 | 10.79 | 18.73 | 57.38 | 3.74 | 11.45 |
