# Supplementary material for: Law of coal caving behind the flexible shield support in pseudo-inclined working face
Source: PLoS One. 2021 Dec 30;16(12):e0261355. doi: 10.1371/journal.pone.0261355 (PMC8717996; doi:10.1371/journal.pone.0261355)
Supplement: S1 File — (ZIP) [file pone.0261355.s001.zip › Supporting information/S3 Table.docx]

**Table 3. Shape, boundary conditions and process parameters of the drawing body.**

| Fall Along the Roof | Fall Along the Floor | | Distance of the Coal Outlet | |
| --- | --- | --- | --- | --- |
| *l_oc_*=*l_hn_*，*l_oc_*=*l_sn_* | Haulage roadway | Working face | Haulage roadway | Working face |
|  |  |  |  |  |
| $\left\{ \begin{aligned} \text{y}^{\text{2}}\text{=}\text{m}\text{h}^{\text{-}\text{n}}\text{x}\text{(}\text{h}\text{-}\text{x}\text{) } \\ \text{x}\text{=}\left( \text{y}\text{+}\text{l}_{\text{oc}} \right)\tan\text{α}\text{ } \end{aligned} \right.$ | $\left\{ \begin{aligned} \zeta\text{=}\text{a}_{\text{1}}\left( \text{ }\frac{\text{φ}}{\text{L}}\text{ } \right)^{\text{b}_{\text{1}}} \\ \text{M}_{\text{f}}\text{ =2ζ } \end{aligned} \right.$ | $\left\{ \begin{aligned} \text{y}^{\text{2}}\text{=}\text{m}\text{h}^{\text{-}\text{n}}\text{x}\left( \text{h}\text{-}\text{x} \right) \\ \text{x}\text{=tanθ}\left( \text{l}_{\text{op}}\text{-}\text{y} \right)\text{ } \end{aligned} \right.$ | $\left\{ \begin{aligned} \text{y}^{\text{2}}\text{=}\text{m}\text{h}^{\text{-}\text{n}}\text{x}\left( \text{h}\text{-}\text{x} \right) \\ \text{x}\text{=}\frac{\text{h}}{\text{2}}\text{ }\text{ } \end{aligned} \right.$ | $\left\{ \begin{aligned} \text{y}^{\text{2}}\text{=}\text{m}\text{h}^{\text{-}\text{n}}\text{x}\left( \text{h}\text{-}\text{x} \right)\text{ } \\ \text{y}\text{=-cotβ}\left( \text{x}\text{-}\frac{\text{h}}{\text{2}} \right)\text{ } \end{aligned} \right.$ |
| $\text{h}_{\text{0}}\text{=}\frac{\text{2}\text{l}_{\text{oc}}\left( \sqrt{\text{1+}\text{m}\text{tan}^{\text{2}} \text{α}}\text{-1} \right)}{\text{m}\tan\text{α}}$ | $\text{h}_{\text{0}}\text{=}\frac{\text{φ}}{\cos\text{α}}\left( \frac{\text{90°-α}}{\text{a}_{\text{1}}} \right)^{\text{-}\text{b}_{\text{1}}}$ | $\text{h}_{\text{0}}\text{=}\frac{\text{2}\text{l}_{\text{op}}}{\text{m}\text{tanθ}}\left( \sqrt{\text{1+}\text{m}\text{tan}^{\text{2}} \text{θ}}\text{-1} \right)$ | $\text{s=}\sqrt{\text{m}\text{h}^{\text{2-}\text{n}}}$ | $\text{s=}\sqrt{\frac{\text{m}\text{h}^{\text{2-}\text{n}}\left( \text{1+}\text{cot}^{\text{2}}\text{β} \right)}{\text{cot}^{\text{2}}\text{β+}\text{m}\text{h}^{\text{-}\text{n}}}}$ |
| $\text{M}_{\text{f}}\text{=}\frac{\text{2}\sqrt{\text{1+}\text{m}\text{tan}^{\text{2}} \text{α}}}{\sqrt{\text{1+}\text{m}\text{tan}^{\text{2}} \text{α}}\text{+1}}\text{l}_{oc}$ | $\text{M}_{\text{f}}\text{=}\sqrt{\text{m}\text{h}_{\text{0}}^{\text{2-}\text{n}}}$ | $\text{M}_{\text{f}}\text{=}\text{l}_{\text{op}}\left[ \frac{\tan\text{α}}{\text{tanθ}}\frac{\left( \sqrt{\text{1+}\text{m}\text{tan}^{\text{2}} \text{θ}}\text{-1} \right)}{\left( \sqrt{\text{1+}\text{m}\text{tan}^{\text{2}} \text{α}}\text{-1} \right)}\text{+1} \right]\sin\text{α}$ | $\text{d=}\sqrt{\text{m}\text{h}^{\text{2-}\text{n}}}$ | $\text{d=}\sqrt{\frac{\text{m}\text{h}^{\text{2-}\text{n}}\left( \text{1+}\text{cot}^{\text{2}}\text{β} \right)}{\text{cot}^{\text{2}}\text{β+}\text{m}\text{h}^{\text{-}\text{n}}}}$ |
| $\text{k}_{\text{cf}}\text{=}\frac{\left( \text{M}\text{-2}\text{l}_{\text{oc}} \right)\sqrt{\text{1+}\text{m}\text{tan}^{\text{2}} \text{α}}\text{+}\text{M}}{\text{2}\text{l}_{\text{oc}}\sqrt{\text{1+}\text{m}\text{tan}^{\text{2}} \text{α}}}$ | $\text{k}_{\text{cf}}\text{=}\frac{\text{M}\text{-}\sqrt{\text{m}\text{h}_{\text{0}}^{\text{2-}\text{n}}}}{\sqrt{\text{m}\text{h}_{\text{0}}^{\text{2-}\text{n}}}}$ | $\text{k}_{\text{cf}}\text{=}\frac{\text{M}\text{sinθcotα}\left( \sqrt{\text{1+}\text{m}\text{tan}^{\text{2}} \text{α}}\text{-1} \right)}{\text{l}_{\text{op}}\text{sin}\left( \text{α+θ} \right)\left( \sqrt{\text{1+}\text{m}\text{tan}^{\text{2}} \text{θ}}\text{-1} \right)}\text{-1}$ | $/$ | $/$ |
