# Supplementary material for: Law of coal caving behind the flexible shield support in pseudo-inclined working face
Source: PLoS One. 2021 Dec 30;16(12):e0261355. doi: 10.1371/journal.pone.0261355 (PMC8717996; doi:10.1371/journal.pone.0261355)
Supplement: S1 File — (ZIP) [file pone.0261355.s001.zip › Supporting information/S4 Table.docx]

**Table 4. Equipment and materials in the working face.**

| Scheme A | | | Scheme B | | |
| --- | --- | --- | --- | --- | --- |
| Equipment and materials | Quantum | Amount | Equipment and materials | Quantum | Amount |
| Individual hydraulic prop | 1540 | 123.20 | Individual hydraulic prop | 180 | 14.40 |
| Articulated roof beam | 1320 | 79.20 | Articulated roof beam | 40 | 2.40 |
| Steel rope | 594 | 0.48 | Steel rope | 880 | 1.58 |
| Liquid injection gun | 12 | 0.24 | Liquid injection gun | 6 | 0.12 |
| Emulsion pump | 2 | 200 | Emulsion pump | 1 | 100 |
| Iron Palm | 1067 | 0.53 | I Beam 11^#^ | 7161 | 154 |
| Round timber | 117 | 0.35 | U clamp, Clamping plate | 7700 | 46.74 |
| Pressure pipe | 560 | 0.11 | Wire netting | 660 | 1.16 |
| Spillplate | 500 | 0.50 | Nut | 15400 | 0.6 |
| Summation | / | 404.61 | Summation | / | 321.00 |
