# Supplementary material for: Law of coal caving behind the flexible shield support in pseudo-inclined working face
Source: PLoS One. 2021 Dec 30;16(12):e0261355. doi: 10.1371/journal.pone.0261355 (PMC8717996; doi:10.1371/journal.pone.0261355)
Supplement: S1 File — (ZIP) [file pone.0261355.s001.zip › Supporting information/S5 Table.docx]

**Table 5. Personnel organization and distribution in the working face.**

| Scheme A | | | | Scheme B | | | |
| --- | --- | --- | --- | --- | --- | --- | --- |
| Activity requirement | Morning | Midday | Evening | Activity requirement | Morning | Midday | Evening |
| Drilling, Blasting | 4 | 4 | / | Drilling, Blasting | 8 | 8 | 8 |
| Monitor, Assist | 3 | 2 | / | Monitor, Assist | 3 | 3 | 3 |
| Stock | 2 | 2 | / | Stock | 4 | 4 | 4 |
| Maintain | / | / | 6 | Maintain | 1 | 1 | 1 |
| Grain | 1 | 1 | / | Forepoling, Installation | 4 | 4 | 4 |
| Complex operation | 39 | 39 | / | Undercarriage | 6 | 6 | 6 |
| Watch keeper | 7 | 7 | / | Planing, Sneak coal | 4 | 4 | 4 |
| Support crew | 4 | 1 | / | Support crew, Drawing | 16 | 16 | 16 |
| Summation | 60 | 6 | 6 | Summation | 46 | 46 | 46 |
