# Supplementary figures and images for: Law of coal caving behind the flexible shield support in pseudo-inclined working face
Source: PLoS One. 2021 Dec 30;16(12):e0261355. doi: 10.1371/journal.pone.0261355 (PMC8717996; doi:10.1371/journal.pone.0261355)

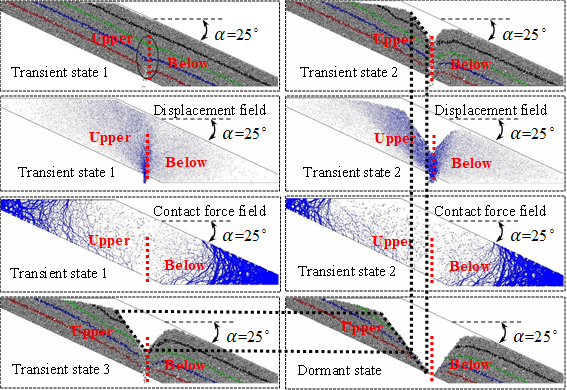

Supplement: S1 File — (ZIP) [file pone.0261355.s001.zip › Supporting information/S10_Fig.tif]

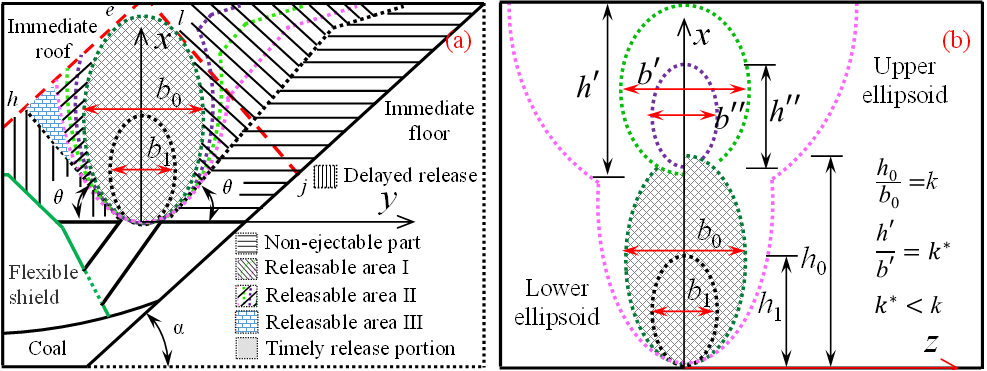

Supplement: S1 File — (ZIP) [file pone.0261355.s001.zip › Supporting information/S11_Fig.tif]

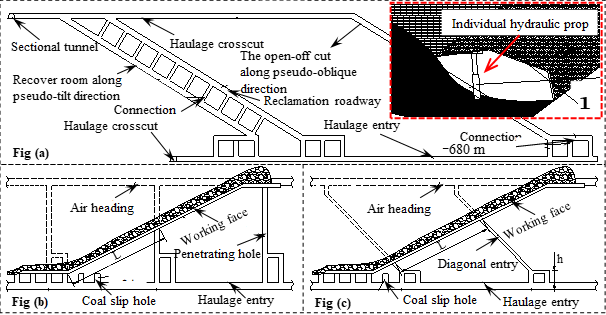

Supplement: S1 File — (ZIP) [file pone.0261355.s001.zip › Supporting information/S12_Fig.tif]

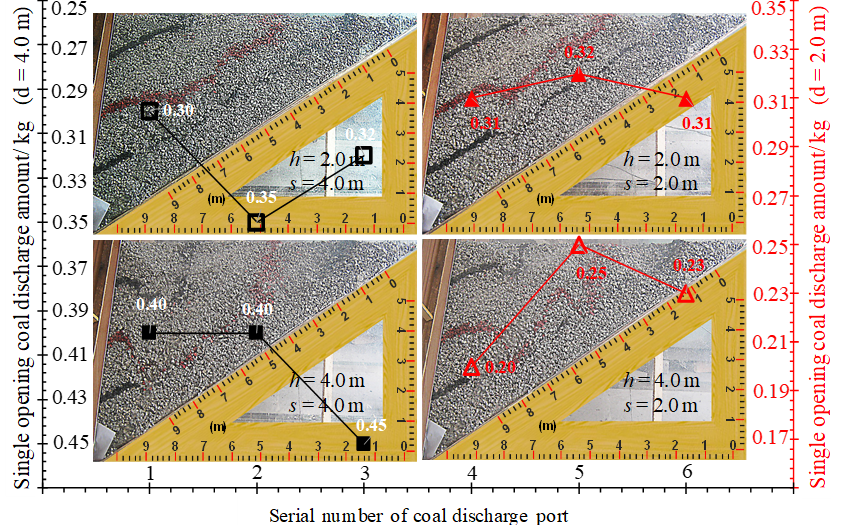

Supplement: S1 File — (ZIP) [file pone.0261355.s001.zip › Supporting information/S13_Fig.tif]

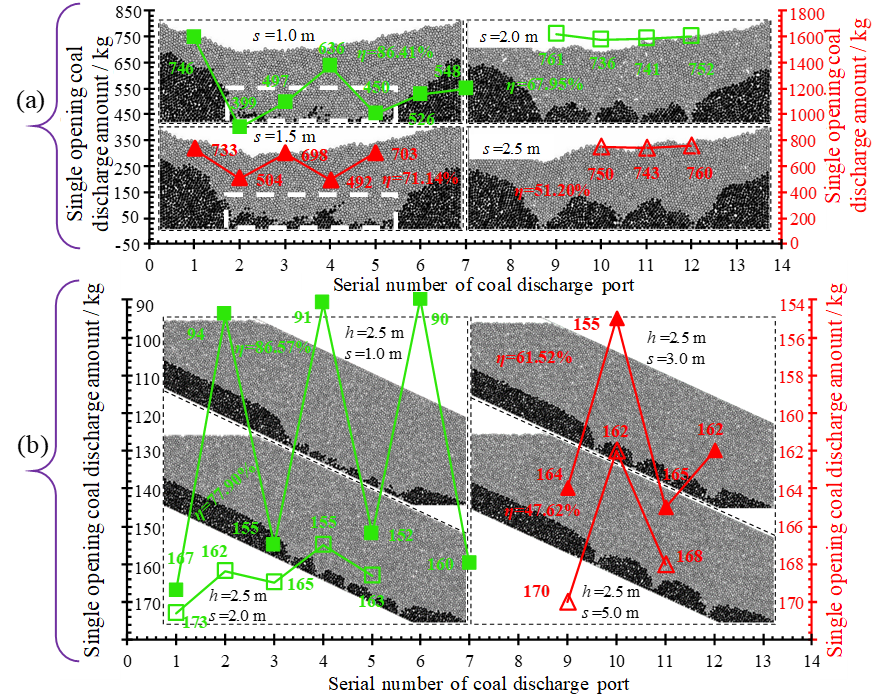

Supplement: S1 File — (ZIP) [file pone.0261355.s001.zip › Supporting information/S14_Fig.tif]

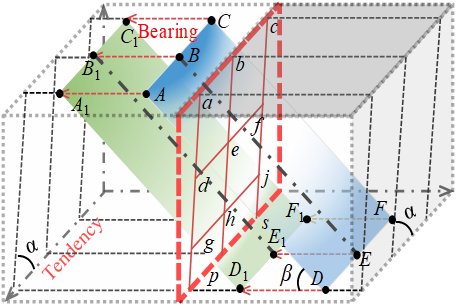

Supplement: S1 File — (ZIP) [file pone.0261355.s001.zip › Supporting information/S1_Fig.tif]

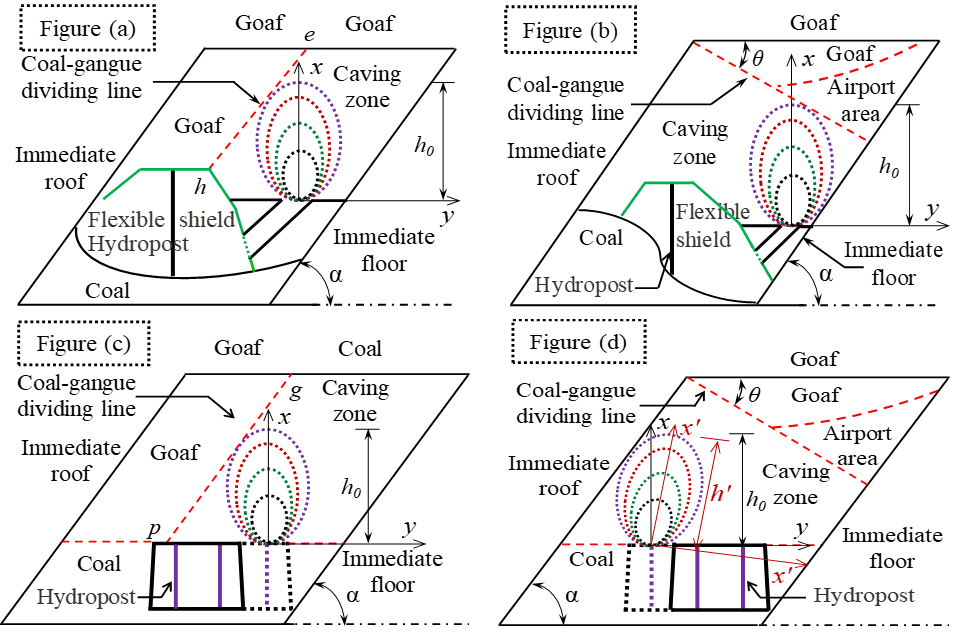

Supplement: S1 File — (ZIP) [file pone.0261355.s001.zip › Supporting information/S2_Fig.tif]

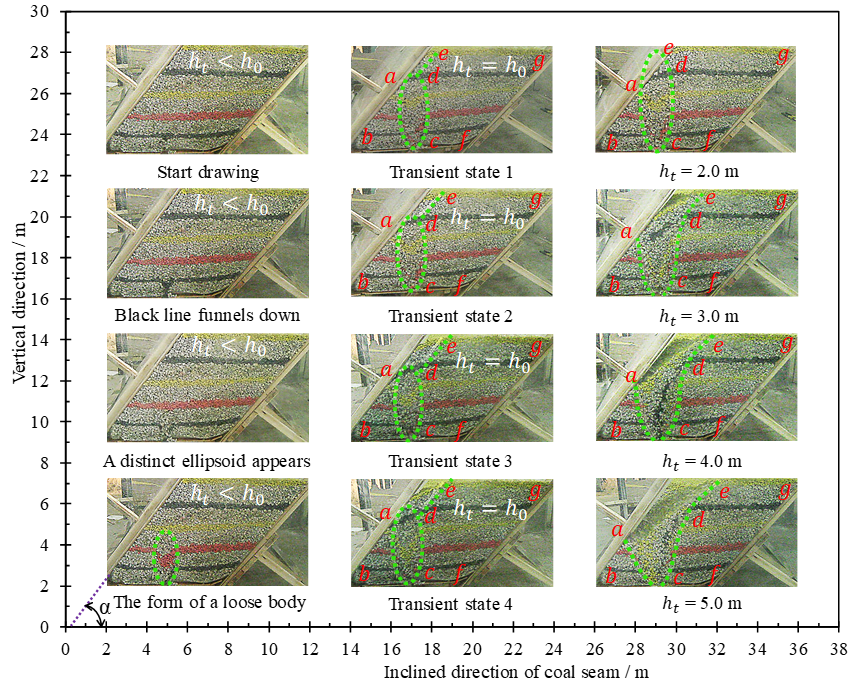

Supplement: S1 File — (ZIP) [file pone.0261355.s001.zip › Supporting information/S3_Fig.tif]

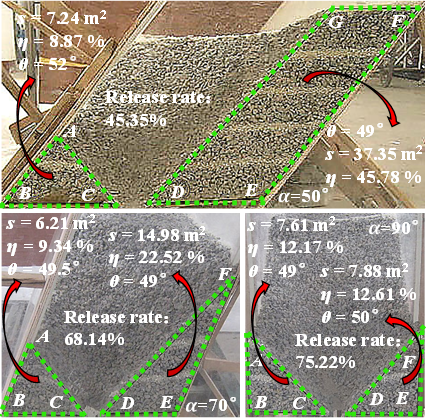

Supplement: S1 File — (ZIP) [file pone.0261355.s001.zip › Supporting information/S4_Fig.tif]

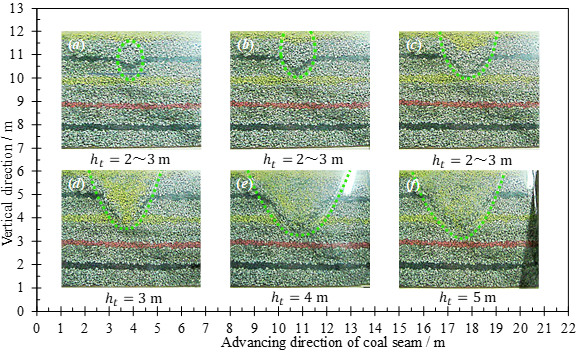

Supplement: S1 File — (ZIP) [file pone.0261355.s001.zip › Supporting information/S5_Fig.tif]

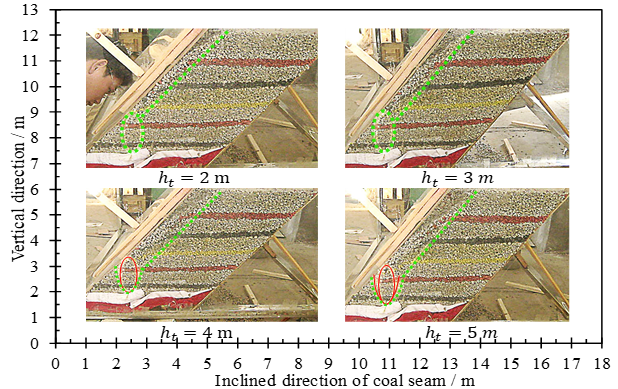

Supplement: S1 File — (ZIP) [file pone.0261355.s001.zip › Supporting information/S6_Fig.tif]

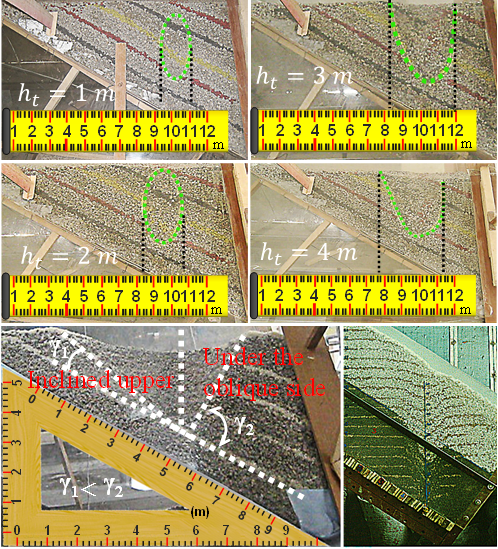

Supplement: S1 File — (ZIP) [file pone.0261355.s001.zip › Supporting information/S7_Fig.tif]

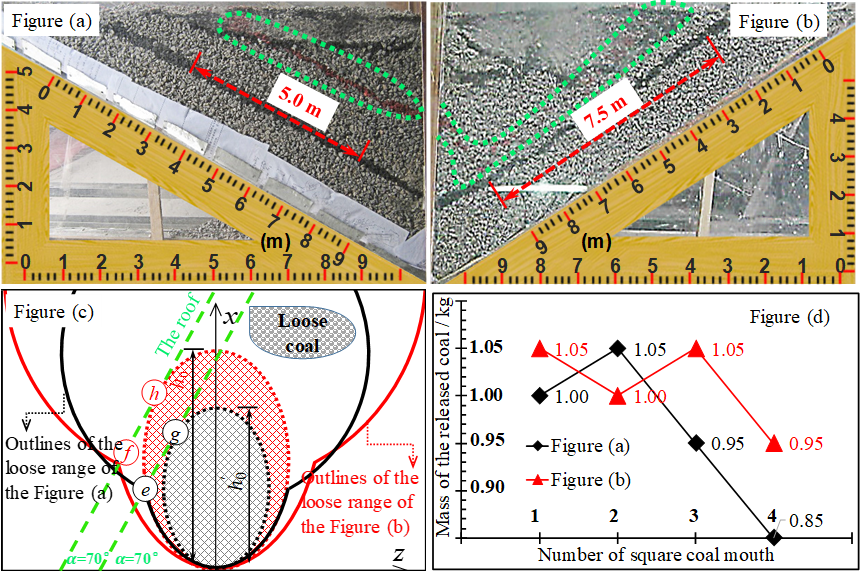

Supplement: S1 File — (ZIP) [file pone.0261355.s001.zip › Supporting information/S8_Fig.tif]

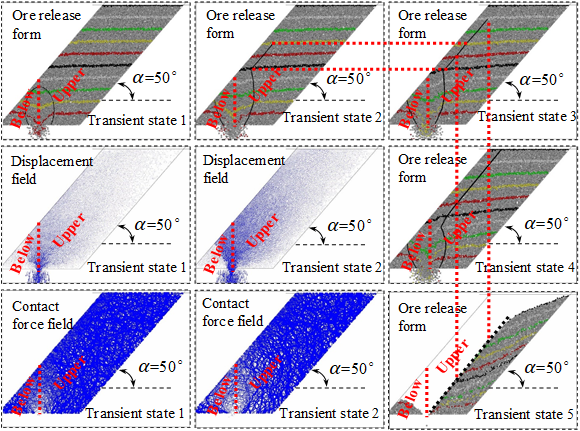

Supplement: S1 File — (ZIP) [file pone.0261355.s001.zip › Supporting information/S9_Fig.tif]
